# A generalizable framework for spatially explicit exploration of soil organic carbon sequestration on global marginal land

**DOI:** 10.1038/s41598-022-14759-w

**Published:** 2022-07-01

**Authors:** Ariane Albers, Angel Avadí, Lorie Hamelin

**Affiliations:** 1 TBI, Université de Toulouse, CNRS, INRAE, INSA, Toulouse, France; 2 CIRAD, UPR Recyclage et risque, 34398 Montpellier, France; 3 Univ Montpellier, CIRAD, Montpellier, France

**Keywords:** Biophysics, Environmental sciences

## Abstract

Land-based CO_2_ removal demands changes in management or new suitable areas to sustainably grow additional biomass without reducing food supply or damaging natural ecosystems. The soil organic carbon (SOC) sequestration pathway is thought to transfer atmospheric CO_2_ into a land unit, through plants, plant residues and other organic solids stored as part of the soil organic matter. No previous study explored SOC sequestration potentials on global marginal land. Here we integrated, into a generalizable modelling framework, the mapping of a set of biophysical (climatic and edaphic) and land conservation constraints to (i) identify suitable matches (i.e. biophysically possible combinations) of target areas with plant species, and (ii) to quantify contributions of pairing to long-term SOC sequestration (2020–2100). The proposed framework represents a refinement to previous mapping exercises, which seldom consider biophysical constraints, soil erosion, plant species tolerances to pedoclimatic conditions, and world protected areas. The approach was tested on marginal lands featuring SOC-deficient stocks (≤ 50 Mg SOC ha^−1^ to 30 cm depth) at 30 arc-sec resolution, consolidated into world regions × global ecological zones based on geo-localised products. The framework was shown to enable better-informed decision-making on interventions at large geographical scales, revealing biophysically realistic options, while management should be determined locally.

## Introduction

A soil organic carbon debt of 116 Pg SOC^1^ was estimated at the global top 2 m of soil, which has increased in the past two centuries due to increasing agricultural and grassland uses. This debt (i.e. the difference between the original pre-agriculture and the current stocks of SOC in exploited soils) demonstrates a strong link between land degradation and SOC losses (e.g. 30–50% of SOC in agricultural mineral soils has been lost due to degradation^2,3^), dependent on the degree of intensity and duration of soil exploitation. It has been recognised that the areas featuring historic SOC losses can now be considered as SOC sinks^4^, with a potential to store two thirds of today’s SOC debt by replenishing SOC stocks^1^.

SOC “sequestration” is considered as a key land-based mechanism, relying on plant photosynthesis to transfer atmospheric CO_2_ into the soil through plants, plant residues and other organic solids stored as part of humus^5,6^. While SOC “storage” refers to the increase in SOC stocks, it does not necessarily rely on atmospheric CO_2_ removal, as external (i.e. imported) inputs such as manure may be added. The former constitutes both a mitigation measure (for climate change but also for ecosystems quality) and a way to induce additional negative emissions (mitigation that would otherwise not have been adopted) as required by the Paris Agreement to limit warming below 2 °C^7^. For instance, the 4 per 1000 initiative, launched at the COP21 Paris Climate Summit in 2015 aims at increasing SOC sequestration through sustainable land management^8^. It is based on the premise that an annual mean SOC increase of 0.4% in the global agricultural topsoil (30–40 cm) would contribute to global sequestration of 2.5 Pg SOC year^−1^^9^—an estimation subject to criticism, due to intrinsic data and model uncertainties^10^; yet representing a clear goal towards climate stabilisation.

In 2020, global mean atmospheric CO_2_ emissions were estimated at 10.2 ± 0.8 Pg C year^−1^^11^. The technical feasibility of SOC sequestration may range as much as between 0.8 and 1.5 Pg SOC year^−1^^3^ and 1.5–3.4 Pg SOC year^−1^^12^—reliant on local land use type (e.g. agricultural), management and restoration (including external inputs) and pedoclimatic conditions, among other factors. Moreover, exploited SOC-deficient stocks < 30 Mg C ha^−1^ are expected to be able to attain high SOC sequestration after adopting best management practices^9^.

The bulk of agriculture’s contribution to SOC storage is influenced by land use management (e.g. addition of organic matter via organic amendments and fertilisers, deployment of improved crop rotations and cover crops), including the cultivation of specific crop types such as perennial and deep rooting species^2,13^. Perennial species have received special attention, as they generally require less soil work, enlarge the C fraction in the soil micro-aggregates, and increase belowground C allocation^14,15^. The extent to which crops in general contribute to SOC sequestration is, however, subject to extensive research^16,17^.

The cultivation of dedicated biomass enabling SOC sequestration and eventually providing feedstock for various economic pathways is intrinsically connected with land demand, and thus with the availability of new areas to grow plants sustainably; i.e. with no adverse effects, for instance, on the SOC debt, food security, ecosystem services, and biodiversity^18^. In the past decade, research has focused on identifying land cover unsuitable for food production but potentially suitable for non-food crops (e.g. bioenergy, biomaterials), defined as marginal land^19^. The expected benefits of exploiting marginal land are wide, ranging from soil quality improvements (e.g. soil fertility, soil structural stability)^20^ to SOC sequestration, through biodiversity conservation^21^ and eventual socio-economic development (e.g. employment, infrastructure, rural poverty alleviation)^22^.

Key types of land classifiable as marginal include degraded and abandoned agricultural lands. Degraded land, which may include soils naturally characterised by low productivity (e.g. natural high salinity soils, or heathlands such as the Mediterranean *garrigue*), has received considerable attention, as a key constituency of marginal land^23^. In particular, research led by the International Soil Reference and Information Centre (ISRIC), throughout various projects such as GLASOD^24^, GLADA^25^ and LADA^26^, applied the use of a remotely sensed global normalised difference vegetation index (NDVI) as a proxy for land degradation due to different causes^27^.

Among the dominant typologies of degraded land, the following FAO classification represents a synthesis of criteria: too cold (polar/boreal), alluvial soil in deserts, too dry, steep lands (dominant slope > 30%), shallow lands, poorly drained, coarse texture, vertisols, infertile (e.g. nutrient-poor), saline/sodic, acid sulphate, and peats (organic soils).The classification is referred to as the FAO agricultural problem land approach^28^, associated with the FAO/UNESCO Digital Soil Map of the World^29^, and now integrated into and superseded by the Harmonized World Soils Database (HWSD)^30^ and into FAO’s Global Agro-Ecological Zones (GAEZ)^31^.

For abandoned agricultural land, another key type of marginal land, extensive research has proposed different definitions and mapping approaches^32–34^. Agricultural land abandonment has been considered to be mainly driven by biophysical constraints, but also by reasons pertaining to farm structure, agricultural viability, as well as to changing population, political regimes, nature conservation and other regional contexts^33,35^. These approaches generically consist of comparing satellite data corresponding to two different periods, and interpreting the temporal land cover differences.

Several studies attempted to quantify and map marginal land use across spatial (global, national, regional and local data) and temporal (historic to current data) resolutions^36^. At the global scale, it has been quantified by mapping current land cover and land suitability indices^37^. However, it has not always been possible to identify the relative importance of agricultural (i.e. abandoned) and non-agricultural land types on the identification of marginal land, because of a combination of factors: analyses based exclusively on (bio)economic drivers, quality of underlying datasets and scales, and lack of detail on current land covers’ spatial (i.e. granularity) and temporal resolutions^18^.

Beyond the challenge of defining and identifying marginal lands, the design of SOC sequestration pathways involving biomass requires the correct pairing of suitable plant species with specific target areas, based on the compatibility of plant species with the target areas’ biophysical characteristics. Such endeavour is not negligible, as demonstrated by the body of research evidence, and recently reviewed at the global scale^18^.

A recently proposed strategy to facilitate global scale climate mitigation^3^, suggests that detailed maps of carbon sequestration potentials including erosion, associated with simple analytical tools, would contribute to supporting the global implementation of SOC sequestration. One of the first examples of such an approach for quantifying global SOC dynamics is that by Morais et al.^38^, which was applied to spatially differentiated unique homogenous territorial units characterised in three main land use classes, namely cropland, grassland and forest land. However, to our knowledge no study has been conducted on SOC sequestration on marginal land at global scale. Here we complement the work by Morais et al. (which focused on non-marginal land) and other approaches focusing partially on marginal land for energy crop production, usually at a regional scale^39,40^.

This study has thus a double purpose: (i) to propose a coherent generalizable framework to facilitate the mapping of marginal lands with low initial SOC stocks (here up to 50 Mg SOC ha^−1^ to 30 cm depth) at global scale and their matching with suitable plant species enhancing SOC sequestration, and (ii) to illustrate the feasibility of the proposed framework by means of a proof-of-concept implementation producing a quantification of SOC sequestration, resulting from suitable matches. Following the hypothesis that global soils with low initial SOC stocks are “non-saturated” and thus feature SOC sequestration potentials^9^, we chose to explore and map global SOC-deficient marginal lands to test this specific condition, yet under consideration of biophysical factors that limit plant growth.

## Results

### Conceptual framework

The proposed framework allows quantifying global SOC sequestration potentials of biomass cultivation on target areas by accounting for both geographic- and plant species-dependent climatic and edaphic limitations. We combined and intersected georeferenced products and automatized the tasks of matching preselected “biopumps” (i.e. plant species featuring SOC sequestration enhancing capabilities and representing a potential source of feedstock for the bioeconomy) to target areas (i.e. aggregations of specific areas of global marginal land, based on ecological zoning). Such matching is established on the pedoclimatic tolerances of the former to the prevailing conditions of the latter, and was followed by a computation of both SOC stock changes due to biomass inputs and SOC losses due to erosion by water, using well-established models.

A simulation time horizon over 2020–2100 was selected in line with IPCC future climate scenarios throughout the twenty-first century for comparison purposes. A R code combined with the data treatment strategy depicted in Fig. 4, constitutes the proposed generalizable framework. The framework is able to produce estimations showing whether more SOC would be sequestered, in the long-term, than it would be lost to rain-driven erosion, if the matching plant species were systematically grown on the identified target areas. To our knowledge, no previous global study on SOC sequestration considered such careful identification of marginal land (i.e. integrating pedoclimatic constraints, plant species tolerances, and land conservation), while balancing both SOC stock changes and SOC losses to erosion.

### Global marginal lands and target areas

To define the extent of marginal land, elements from various studies^18,39,40^ were combined to define marginal lands as land covers that are currently unused by agriculture due to an aggregation of socio-economic and biophysical constraints, or human-induced land degradation, but which could potentially be suitable for sustainable biomass production. Identified marginal lands (in total 2714 Mha) in this case-study consisted of non-agricultural land covers: i.e.: bare land (74.49%), sparsely vegetated areas (25.39%), including abandoned agricultural land (0.14%). These areas are presented in Supplementary Results, Fig. S1 for near-present conditions with SOC stocks divided into five SOC classes up to 50 Mg SOC ha^−1^ (to 30 cm depth) following increments of 10 Mg SOC ha^−1^. Additional intermediate maps are also presented in Supplementary Results, depicting marginal land (Fig. S2), examples of agricultural land abandonment (Figs. S3–S6) and the criteria leading to the identification of target areas (Fig. S7–S10) under the retained constraints (e.g. limiting initial SOC stock to 50 Mg ha^−1^, excluding protected areas, considering pedoclimatic constraints to plant growth, and consolidating target areas into global climate zones and world regions).

**Figure 1:**
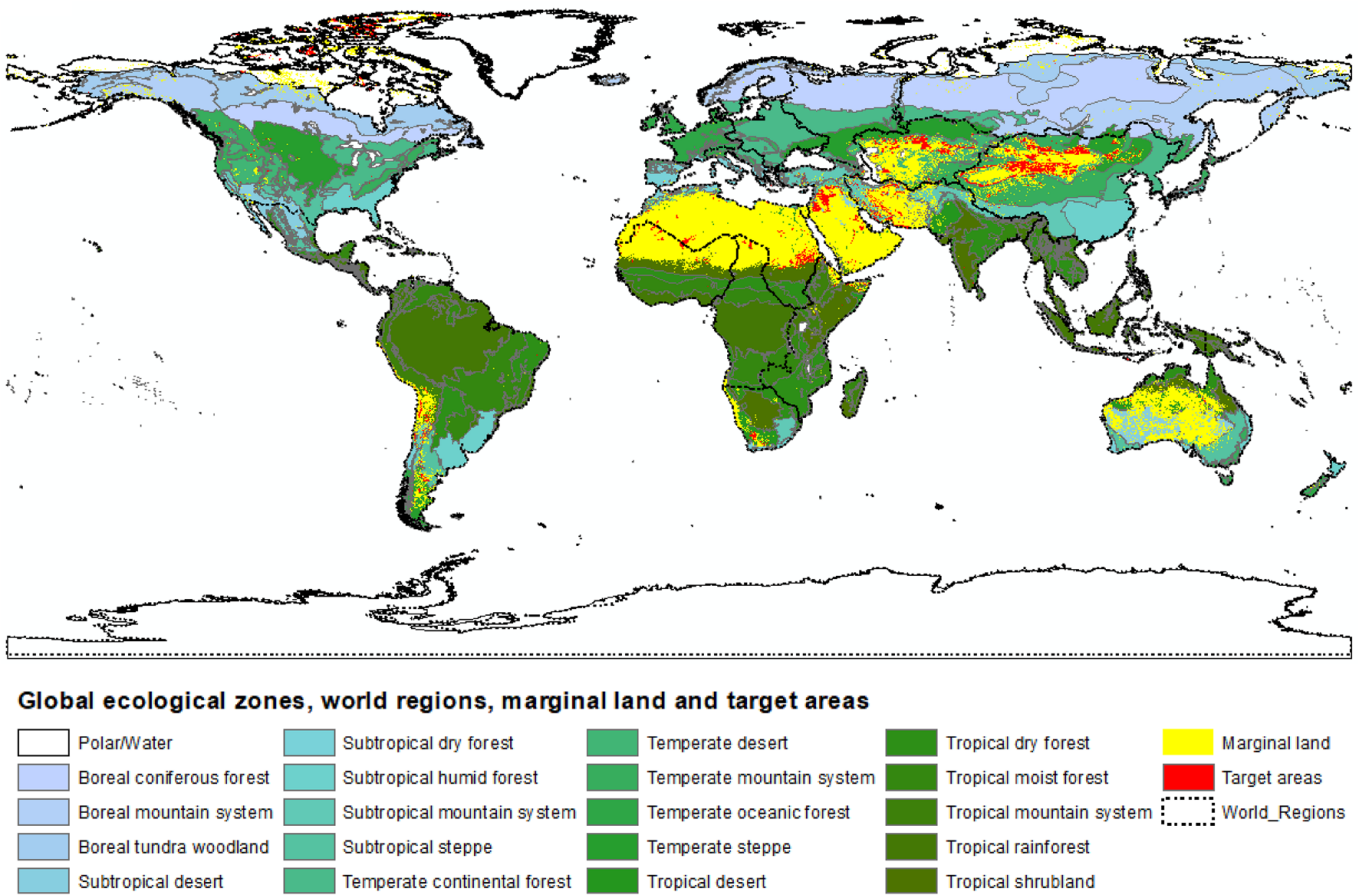
Identified target areas on marginal lands here containing up to 50 Mg SOC ha^−1^ (to 30 cm depth) per global ecological zone and world region.

Figure 1 illustrates the resulting consolidated target areas, determined by geographies established as the combination of climate zones defined in FAO’s Global Ecological Zones (GEZ)^41^ and geo-political world regions. Overall, 27.2 Mha of land was identified as target areas based on 116 possible combinations of 21 world regions and 19 GEZ (here excluding water and polar) (Supplementary Methods, Tables S5 and S6). The identified target areas represent 1% of the initially defined SOC-deficient global marginal lands due to biophysical (e.g. pedoclimatic constraints to plant growth) and land conservation limitations.

**Figure 2:**
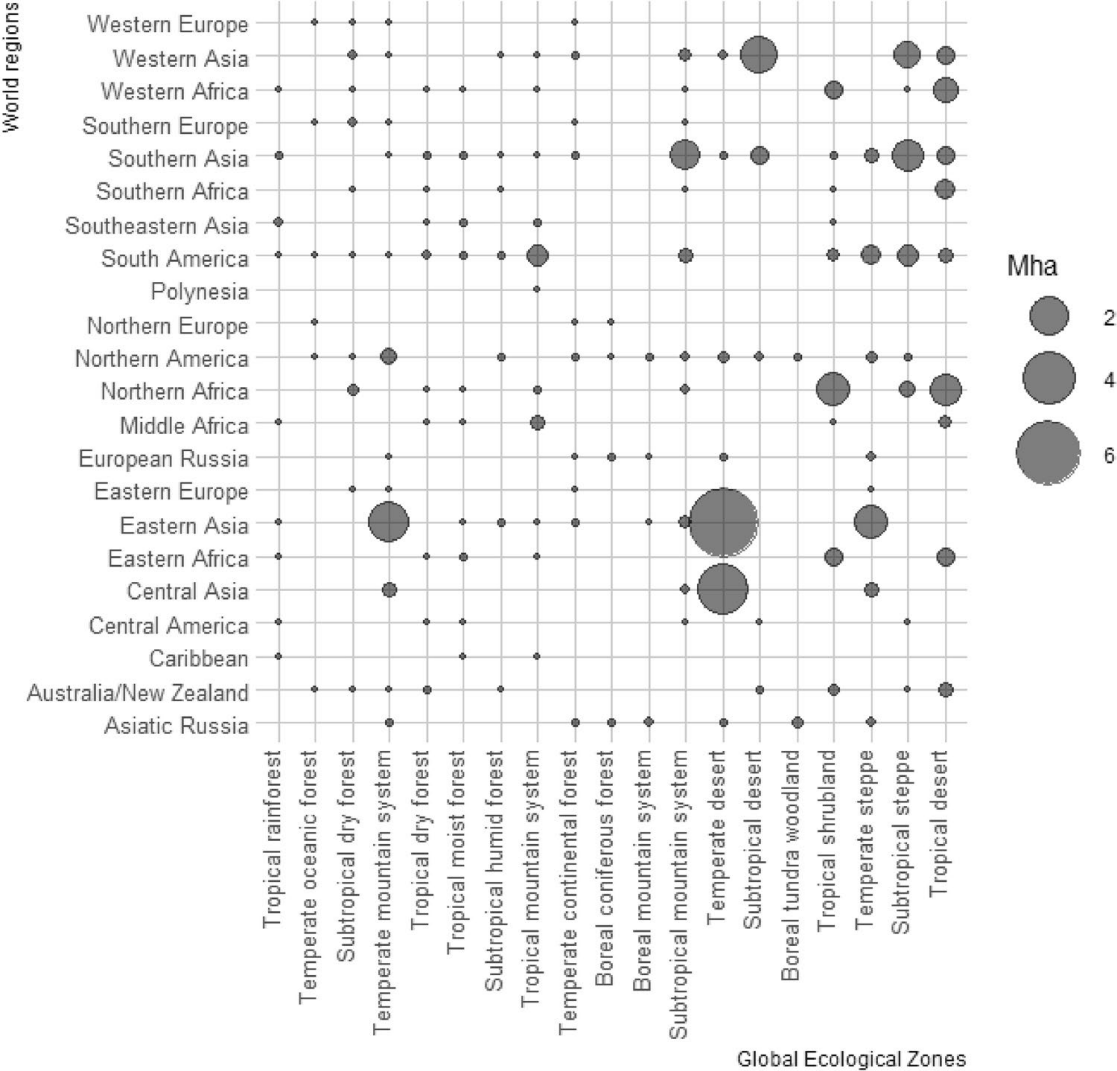
Relative geographical concentration of identified target areas.

The majority of target areas were found in Asia (Eastern, Central, Southern, and Western), Northern Africa, and South America (Fig. 2), corresponding to GEZ in the Temperate, Tropical and Subtropical desert and mountain systems, as well as Temperate and Subtropical steppe. Europe accounted for 0.1% of all target areas, largely present in the Southern Europe Region concordant with GEZ Subtropical dry forest. All values per world region and GEZ are listed in the Supplementary Results, Table S1.

### Suitable “biopumps” with soil carbon organic sequestration potentials

Initially, 50 biopumps were preselected by scoring and ranking plant species on SOC sequestration enhancing capabilities, primary yield productivity, and marginal land adaptability. Preselection criteria and approach used are detailed in Supplementary Results, Table S2, including SOC sequestration potentials in Table S1 and Fig. S10. In total, 432 plant species were associated to the 50 preselected biopumps based on FAO’s ECOCROP database^42^. Datasets on the plant environmental requirements (i.e. climatic and edaphic conditions) were retrieved from the database to further assess the suitability of plant species on target areas. All datasets on plant species tolerance are available from a data repository^43^. The eventual introduction of invasive species in the receiving ecosystems was not considered beyond the exclusion of world protected areas.

The matching exercise resulted in 561 viable combinations (out of 50 112 theoretically possible matches) of target areas (116 GEZ × world regions) × biopumps (432 plant species), where 27 biopumps (associated to 56 plant species) were compatible with 12 world regions and 12 GEZ. The total area with cumulative net SOC stocks > 0 Mg ha^−1^ in the simulation end-year 2100 (i.e. the sequestered SOC is larger than the eroded SOC, implying either accumulation or a reduction of the initial SOC without reaching zero), comprised 0.55 Mha, representing about 2% of the initially identified target areas (27.2 Mha in total). The largest concentration in area was found in Western Africa × Tropical shrubland (0.30 Mha), Australia/New Zealand × Tropical desert (0.15 Mha), Australia/New Zealand × Tropical shrubland (0.04 Mha) and South America × Tropical dry forest (0.02 Mha).

**Figure 3:**
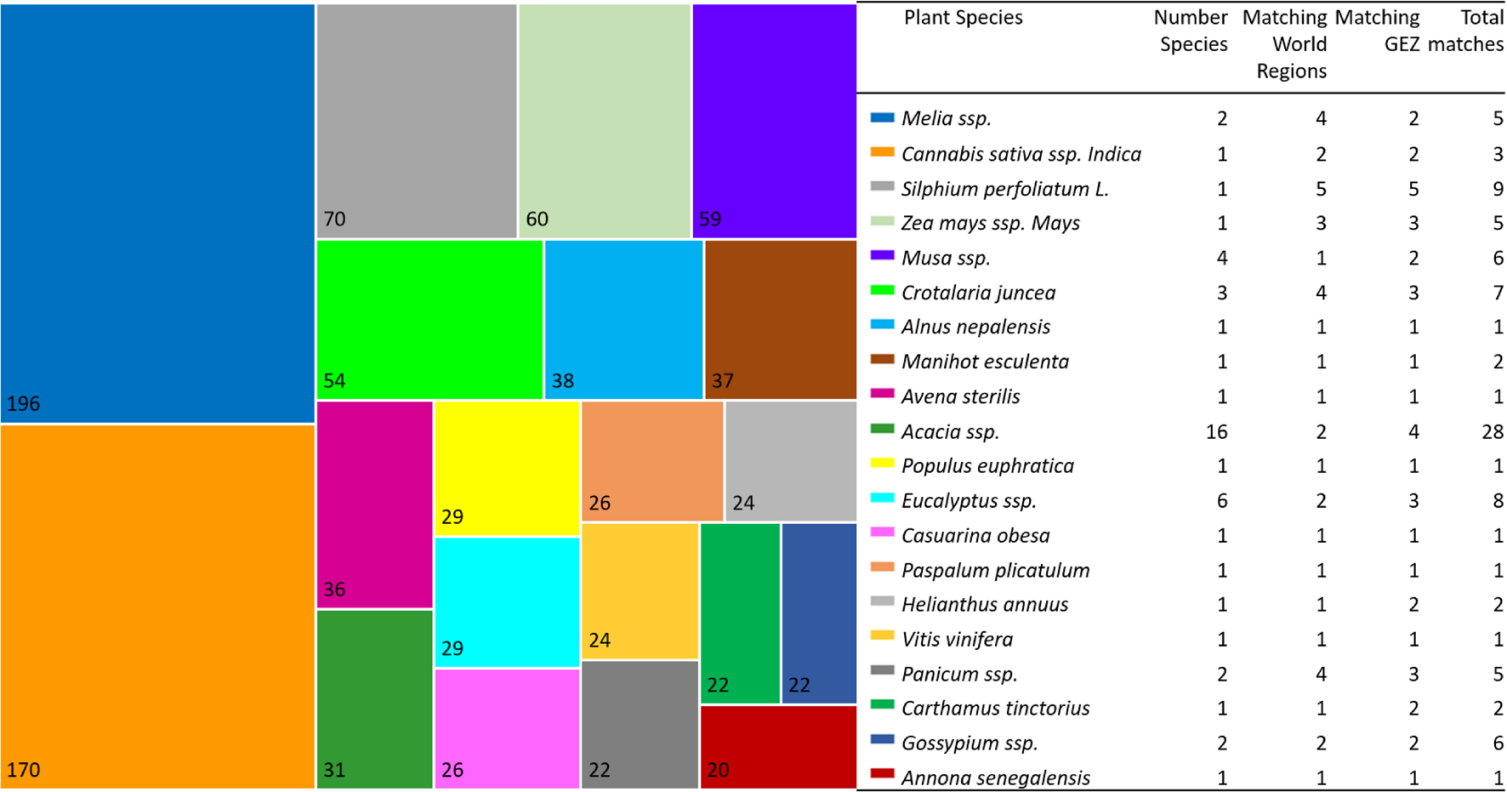
Top biopump soil carbon sequestration potentials, prior to erosion (number in boxes in Mg SOC ha^−1^ in year 2100) and preliminary compatibility with corresponding number of plant species, matching world regions and global ecological zones (GEZ), as well as total number of matches per target area.

Figure 3 provides an overview of the top 20 plant species with matching-related metadata. The top 5 biopumps with the highest SOC sequestration potentials in 2100, prior to the consideration of erosion, were neem (*Melia azedarach*), hemp (*Cannabis sativa* spp.* Indica*), cup plant (*Silphium Perfoliatum* L.), maize (*Zea mays* ssp. Mays)*,* banana (*Musa* ssp.), with 196, 170, 70, 60, 59 Mg SOC ha^−1^, respectively. The biopumps with the highest number of pairings per region and/or GEZ, were acacia (*Acacia* ssp.), cup plant (*S. perfoliatum*), eucalyptus (*Eucalyptus* ssp.), sun hemp (*Crotalaria juncea*), banana (*Musa* ssp.), and cotton (*Gossypium* ssp.).

### Best-case combinations with the highest sequestration potential

Overall, 112 possible target area × biopump combinations (i.e. 20% of all viable matches) feature cumulative net SOC stocks > 0 Mg ha^−1^ in 2100. About 24% (i.e. 26 possible matches) represent one biopump per target area. The selection of one biopump per target area (world region × GEZ) yielding the highest net SOC stock in 2100 are referred to as a “best-case combination”. In total, 11 biopumps (associated with 14 species) were identified to yield the highest net SOC stock in 2100, whereas the most representative in number of matches were cup plant (*S. perfoliatum*, 9 matches) followed by neem (*Melia* ssp., 3 matches), and hemp (*C. sativa* ssp.* indica*, 3 matches). Data of the 26 best-case combinations are presented in Supplementary Results, Table S2.

A comparison of SOC stocks for the baseline year 2020 (referred to as “initial SOC”) with the final state of the simulation in 2100 (referred to as “final SOC”) has shown, for these best-case combinations, that the observed SOC stock level increases compared to initial SOC are significant (p-value < 1%). Overall net SOC stock potentials (final SOC minus eroded SOC) in 2100 ranged between 2.57 and 158.04 Mg ha^−1^ (equivalent to about 0.03–1.98 Mg ha^−1^ year^−1^).

Top 5 best-case combination were Australia/New Zealand × Tropical dry forest × *M. azedarach var australasica*, Northern Africa × Tropical dry forest × *M. azedarach*, Eastern Africa × Tropical dry forest × *C. sativa* ssp.* indica*, Central America × Tropical dry forest × *C. sativa* ssp.* indica*, and Eastern Africa × Tropical mountain system × *C. sativa* spp.* indica*, with net SOC stocks at 158.04, 144.87, 135.57, 118.50, 74.56 Mg ha^−1^, representing 93%, 98%, 80%, 87%, 50% of final SOC prior to erosion, respectively (Supplementary Results, Table S2). There were exceptions of best-case combinations, namely the matches: Australia/New Zealand × Tropical desert × *A. erioloba*, Middle Africa × Tropical moist forest × *P. plicatulum*, South America × Tropical dry forest × *Miscanthus* ssp., Australia/New Zealand × Subtropical steppe × *J. curcas*. These four situations, despite reaching net SOC > 0 Mg ha^−1^ in 2100, corresponded to a reduction of the initial SOC stock, respectively by 16%, 42%, 43%, 54%, as compared to the final SOC stock (Supplementary Results, Table S2); demonstrating that the implementation of biopumps would generate a loss of initial SOC stocks (in addition to losses from water erosion).

Spatially dependent soil erosion affected the net SOC potentials significantly (p-value < 1%). Long-term improvements in final SOC were not achieved for most originally identified 561 viable combinations. From the resulting best-case combinations, the most affected target areas by water erosion were: Tropical moist forest in South America, Temperate oceanic forest in Australia/New Zealand, Tropical dry forest in South America, and Tropical moist forest in Western Africa, where the resulting SOC losses represented respectively 91%, 88%, 88%, 86% of the final SOC (Supplementary Results, Table S2). About one third of the best-case results showed negligible SOC losses by erosion (up to 7% of final SOC values before erosion) located in European Russia (and Boreal coniferous forest and Boreal mountain system), Western Europe (Temperate mountain system) and Northern Europe (Temperate continental forest, Temperate oceanic forest and Boreal coniferous forest), Australia/New Zealand (Tropical dry forest and Temperate oceanic forest).

## Discussion

The framework design is generic enough to be implemented in other settings, regarding target area definition, plant species of interest, scopes (e.g. geographic boundaries), as well as temporal and spatial resolutions. The provided R script in Ref.^43^ is usable in different situations as long as the required input data is organised in the prescribed way. Moreover, the framework is flexible to accommodate different SOC sequestration and erosion models (e.g. by wind), depending on the required outputs and data availability. For our example, we selected the monthly time-stepped RothC model (detailed in “Methods” section), being an appropriate SOC model due to its: (i) demonstrated performance to replicate observed SOC changes in validation experiments, as compared with other models (described in Supplementary Methods), (ii) applicability to a wide range of world climates and regions in combination with GIS products^4,38^; (iii) recognition, being recommended as a standard spatialized SOC model at a 30 arcsec resolution by the FAO^44^.

As a proof-of-concept we applied the framework on SOC-deficient global marginal lands under well-defined criteria and were able to identify suitable plant species delivering highest net SOC sequestration on marginal lands, thus showing the feasibility and interest of the proposed framework to produce immediately informative results. These results could not be compared with those of previous studies because no previous research, to our knowledge, has focused on the same definition of marginal lands (featuring SOC erosion, land conservation areas, and pedoclimatic constraints to plant growth).

The climatic and edaphic characteristics of the target areas were averaged at the world region × GEZ scale. Despite the loss of detail, we considered that the consolidated target areas were the more suitable geographical resolution to explore biopump compatibility to marginal land at a manageable scale. Modelling implications of different geographical resolutions, however, requires further assessment. Furthermore, the projected long-term SOC changes were based on near-term climate data, i.e. disregarding climate change effects on SOC stocks dependent on temperature, precipitation and evapotranspiration variables. Both the uncertainties associated to the consolidation of target areas and future climate trajectories (e.g. as defined in the 6th Climate Model Intercomparison Project, CMIP6^45^) are addressed in an upcoming publication by our team.

We identified abandoned agricultural land, as a key component of marginal land, of about 3.76 Mha for SOC stocks ≤ 50 Mg ha^−1^ (to 30 cm depth) over the years 2010–2018 (about 0.47 Mha year^−1^) based on ESA CCI^46^. A global historic land use comparison over the period 1700–2000^47^ determined permanent cropland conversions into other land covers of about 210 (about 0.7 Mha year^−1^) and 269 Mha (0.9 Mha year^−1^) based on the datasets HYDEv3.0^48^ and SAGE^49^ respectively. A recent study^35^ quantified the world’s current extend of land use change and developed a model called HILDA+ to provide annual harmonised global land use data with national inventories at near-present. Cropland use changes were identified at 1029 Mha over the period 1960–2015 (about 0.19 Mha year^−1^) with HYDEv3.2^50^ and 1035 Mha over the period 1960–2011 (about 0.20 Mha year^−1^) with SAGE^49^. A comparison of the former and the later with HILDA+ over the same periods showed higher results by 31% and 24%, respectively. The annualised values are comparable among different studies with different temporal resolutions; however, it generates uncertainty in the interannual variability as well as in scope and definition of abandoned agricultural land vs. cropland use change.

A key hypothesis for high initial SOC losses, despite biopump implementation, was that very strong seasonal precipitation, coupled with incomplete protection of the soil by canopy (expressed as the cover management factor in the erosion model) may lead to these effects. Another hypothesis, for the global case study selection, was that only SOC-deficient land covers are interesting for biopump implementation, as they have not reached SOC saturation and are likely to attain higher sequestration potentials^51^. However, SOC-deficient soils with < 0.5% and < 0.75% organic carbon content (equivalent to 21 and 31.5 Mg SOC ha^−1^ respectively to 30 cm depth^52,53^)may feature severe to sub-severe soil fertility deficiency^54^.

We disregarded the (sub-)severe soil fertility constraints for this exercise by cross-referencing global land covers with global SOC stocks ≤ 50 Mg ha^−1^ (up to 1.2% organic carbon content to 30 cm depth). This soil constraint might even further reduce the identified target areas. However, we excluded it because the residual biomass, as input to the soil, contributes to soil fertility and other management practices might further improve the soil quality^9^. Any land use management (or change) influences the evolution of SOC, which need to be specified in the soil model. In our example, we have not considered additional management practices other than the inclusion of plant-based C-inputs. Cultivation practices in terms of resource requirements (e.g. nutrient supply, irrigation), including best management practices (e.g. organic amendments and fertilisers, cover crops), should be considered in more detailed assessments, as they have been shown to influence SOC sequestration rates^9^. Other specific agronomic requirements (e.g. to prioritise only low-input species on marginal land), as well as ecological impacts and invasion risks (of so-called alien or invasive species), need further assessment.

Moreover, RothC is a non-saturating SOC model^55^, yet deemed to yield accurate predictions in cases when C-inputs are “low”^56^ (e.g. in the absence of organic fertilisation). A further refinement in the framework would involve adding a C-saturating model to better account for management practices, including organic fertilisation^55,57,58^.

About 13% of all inventoried plant species were suitable for the identified target areas, yet representing about 54% of the preselected biopumps. The preselection criteria alone were not a qualification for SOC improvements and biomass productivity. The study has demonstrated that the net sequestration is a result of a combination of biophysical factors (e.g. soil, terrain and climate types, rain erosion, etc.), and that the performance of one crop on one target area is not the same on another. Several other elements would require consideration, such as local environmental challenges (e.g. compaction and low water retention, limited nutrients, low organic matter and potential phytotoxicity, weed and pests, etc.), trade-offs with competing land uses (e.g. forage and livestock, housing, conservation, recreation, etc.), and socio-economic market interferences (e.g. disruption of value chains or people’s livelihoods).

In conclusion, for robust estimation of SOC turnover in marginal lands, erosion and pedoclimatic limitations to plant growth should be considered. Moreover, marginal lands should be considered for SOC sequestration initiatives as an untapped resource, beyond agricultural/forestry land through management, despite potentially low productivity.

## Methods

**Figure 4:**
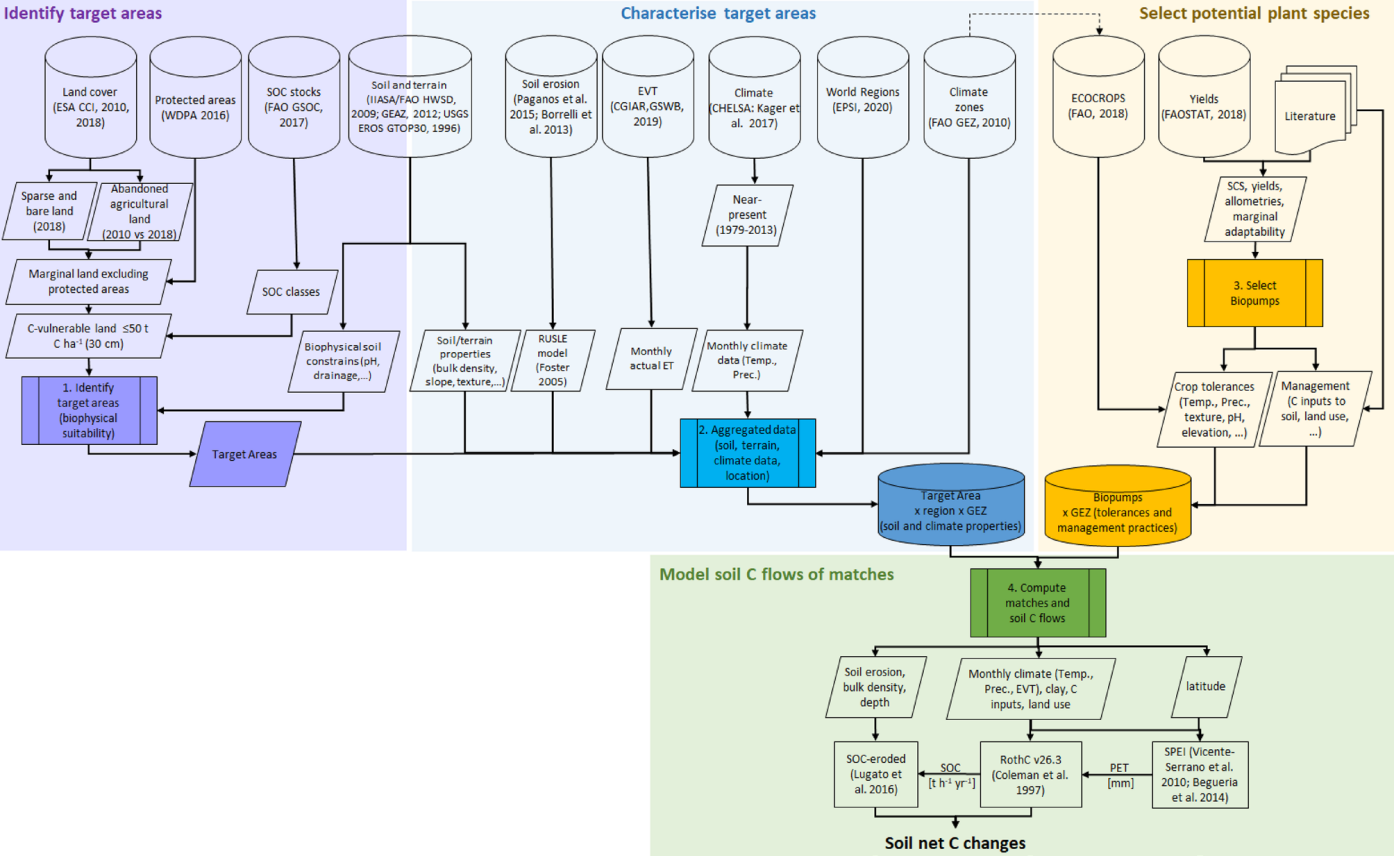
Stepwise framework and data sources for our case study. *Temp.* temperature, *Prec.* Precipitation, *EVT* evapotranspiration, *SPEI* standardised precipitation evapotranspiration index, *PET* potential evapotranspiration, *SOC* soil organic carbon.

We developed a framework to explore SOC sequestration potentials by various plant species and categories, and applied it to identifying and consolidating global target areas delimited by geopolitical and environmental boundaries. The proposed framework relies on the use of georeferenced products, corresponding to the needs of macro-level global models. It was structured in four main steps (Fig. 4):

1. identifying target areas corresponding to land covers of interest, while considering biophysical constraints to biomass production and land conservation (in our example: global marginal land);
2. characterising target areas by their pedoclimatic and terrain conditions, and consolidating them into the required level of aggregation/granularity (in our example: consolidated target areas defined by geopolitical world regions and geoecological zones),
3. selecting plant species or groups of species (in our example: promising biopumps) and determining their environmental requirements/tolerances, and
4. matching target area × biopumps as determined by the compatibility of plant species to target areas’ biophysical characteristics, and modelling soil C flows of the resulting target areas-biopump pairs.

The full list of exploited data and sources of georeferenced data is presented in the dataset^43^ and Supplementary Methods, Table S1.

### Identification of marginal land (steps 1)

Global marginal lands were mapped at a 30 arcsec (1 km) resolution, using a geographic information system (ArcGIS v10.6). We studied different definitions of marginal land to segregate land types into agricultural (potentially suitable for food production historically, currently or in future) and non-agricultural (unsuitable/unfavourable for food production). The selection was based on a combination of various criteria of soil constraints to biomass (plant-based) production from key marginal land studies (detailed in Supplementary Methods, Tables S2 and S3) to ensure that their associated biophysical constraints would be compatible with the environmental requirements for plant growth, and that chosen areas correspond to a land cover category that, if changed, would not contribute to further environmental degradation (including SOC losses). In our example, we followed five sub-steps to identify marginal lands:

In step 1.1, we explored the global land cover (LC) map by the European Space Agency Copernicus Climate Change Initiative (ESA-CCI) for the year 2018 at a 300 m (9.7 arcsec at the equator) resolution, which features 22 LC classes defined in the FAO Land Cover Classification System^59^ (see Supplementary Methods, Table S4). We retained bare and sparsely vegetated (< 15% of vegetation) areas, as all other LC classes likely feature food/feed production (e.g. irrigated or rainfed cropland, natural grasslands), densities of vegetation that would risk severe damage to natural ecosystems and SOC losses from land use change (e.g. forests and shrubland), or are vulnerable to crop growth due severe biophysical constraints (e.g. lichens and mosses).

In step 1.2, we identified recent abandoned agricultural land, understood as the share of marginal agricultural land that is unused or underutilised. The extent of abandonment was associated with land cover change from previous irrigated or rainfed cropland to other LC classes, by comparing ESA-CCI^46^ georeferenced products from the years 2010 and 2018. We considered, elaborating upon the definition from Van Asselen and Verburg^60^, a transition to mosaic cropland/natural vegetation (complemented with mosaic cropland/natural vegetation to semi-natural), grasslands, sparse vegetation, bare areas, mosaic herbaceous cover or shrubland.

In step 1.3, we refined the previous filter by analysing the global LC regarding SOC stocks [Mg ha^−1^] in topsoil (≤ 30 cm), based on the FAO’s Global Soil Organic (GSOC v1.5) map^61^ for the year 2017, and retaining SOC-deficient areas featuring up to 50 Mg ha^−1^.

In step 1.4, we filtered out areas corresponding to geographically defined protected areas, particularly important for biodiversity and ecosystem services, covered in the World Database on Protected Areas (WDPA v1.6) map, a georeferenced product of the UN Environment Programme World Conservation Monitoring Centre (UNEP-WCMC)^62^.

In step 1.5, we identified the biophysical constraints associated with the lands satisfying the previous two conditions, in order to discard areas featuring (sub-)severe soil and terrain restrictions that would exceed the tolerances of potential plant species. These constraints correspond to those associated with degraded lands in the georeferenced FAO Dominant Type of Problem Lands product for a generic perspective, complemented by the Harmonized World Soil Database (HWSD v1.21)^30^ on soil properties.

### Characterisation and consolidation of target areas (step 2)

The identified marginal lands were further characterised with their edaphic and climatic conditions for an iterative refinement in finding possible matches with biopumps’ environmental tolerances, as well as to inform SOC models. In our example, we used the following georeferenced products:

- Soil properties (e.g. texture, clay content, pH, bulk density, depth) and terrain specification (slope and elevation) were obtained, respectively, from HWSD v1.21^30^ and GMTED2010^63^ at a 30 arcsec resolution.
- Near present climate data (monthly temperature and precipitation, and evapotranspiration) were retrieved from the Climatologies at High resolution for the Earth’s Land Surface Areas (CHELSA v1.2)^64^ at 30 arcsec for the years 1979–2013.
- Geographic boundaries were established with a Global Shapefile from the global administrative areas (GDAM v4) database^65^. Layers for world regions (represented by the boundaries of 25 commonly recognised regions (Supplementary Methods, Table S5), as well as latitudes and longitude grids at 30 degrees, were retrieved as Esri ArcGIS Data and Maps products.
- Climate zones were classified as in FAO’s Global Ecological Zones (GEZ)^41^, consisting of 19 classes based on essential biological boundaries, i.e. with relatively homogeneous vegetation physiognomy (Supplementary Methods, Table S6).

Once all individual (contiguous pixels) marginal lands were characterised, it was necessary to consolidate them into larger target areas, according with the desired scope and granularity. In our example, we consolidated all marginal lands within the same GEZ (e.g. Tropical shrubland) and geo-political world region (e.g. West Africa), coarsely following^66^.

### Identification and characterisation of plant species (step 3)

A preselection of potential biopumps was performed and the plant data recorded into a database to evaluate their suitability to grow on identified and consolidated target areas based on their pedoclimatic requirements, and to inform the SOC model (e.g. plant-based organic C-inputs). Initially a list of 164 herbaceous and woody plants were considered, and data compiled from diverse data sources specific to agricultural perennial^67^ and lignocellulosic bioenergy^68^ crops, including all 68 industrial crops initially considered in the EU H2020 MAGIC project (www.magic-h2020.eu/). The preselection was performed via a semi-quantitative multi-criteria analysis by scoring and ranking SOC sequestration performance from above and below-ground C-inputs to the soil, feedstock productivity, and marginal land suitability (detailed in Supplementary Methods, Table S7).

Data on the pedoclimatic tolerances provided in Ref.^43^ for the matching were collected from the FAO’s ECOCROP database^42^, per biopump, for the following variables: temperature [°C], precipitation [mm], pH, soil texture (coarse, medium, fine), altitude [m], and global climate zones. Other essential soil properties (e.g. soil drainage, rooting deep) were previously considered as limiting factors for determining target areas suitable for biomass cultivation. The ECOCROP climate zone classification system based on Köppen^69^ was harmonised with the FAO’s GEZ^70^ used for target areas consolidation, to enable the matching (harmonisation detailed in Supplementary Methods, Table S6). Moreover, several plant species with the same common name were recorded, showing different tolerances, to ensure a broader initial pool for the matching exercise, and because species specifications were lacking for some of the previously used datasets.

The land use class of biopumps was classified into three types based on the life form described in retained datasets^42,67,71^: grass (e.g. bahiagrass, hemp, miscanthus, banana, bamboo, etc., including small woody or herbaceous shrubs (e.g. blueberry, etc.), crops (e.g. sugar cane, maize, loofah, etc.), and trees (e.g. short rotation coppice, orchards, tree-nuts, etc., as well as woody shrubs that are tree-like, e.g. due woody stem, lifetime, size and physiognomy of the plant). Uncertainties may have accumulated where the boundaries between grass and woody species are not clear.

Data provided in Ref.^43^ on plant organic C-inputs [Mg ha^−1^ year^−1^] were computed by the fractioning and C partitioning approach^72^, individually for above- (product, stem, and leaves) and below- (roots) ground compartments and C contents per fraction [%]^73^. The total C-inputs were resolved by partitioning the C per fraction to the soil, depended on the lifecycle (annual or perennial) and lifetime in years. For perennial species several estimates are required, as variations arise from annualising the aboveground C-inputs (e.g. whether the leaves are deciduous or evergreen or the stem is considered a bioeconomy feedstock). Roots, on the contrary, remain over the entire rotation length in the soil. We adopted a linear approach by dividing the total C root by the lifetime in years, here assuming an uneven-aged approach^74^.

### Matching of target areas and plant species and computation of net sequestration per target area × biopump (step 4)

Once both target areas and a list of candidate plant species were identified, it was necessary to determine matches that would be, ad minimum, biophysically possible. A clear way to achieve such pairings is to compare the pedoclimatic conditions prevailing in the target areas with those of the plants.

We developed a R (R Core Team^75^) engine provided in Ref.^43^ to firstly identify the corresponding matches between potential biopumps and target areas, and subsequently run the models for both SOC stock changes and SOC losses to erosion per matched target area-biopump pair up to the year 2100. A match took place when the tolerance ranges of biopumps were within the values associated to the target areas (e.g. temperature, pH, elevation, climatic zone compatibility, etc.). The resulting combinations contain all the information gathered from the previous modules, which is a unique set of input data required to initiate the SOC model and compute the associated SOC stock changes.

We used the monthly time-stepped and processes-based Rothamsted C (RothC) model v26.3^76,77^ to compute SOC sequestration dynamic (described in Supplementary Methods, including a model comparison in Table S8). RothC, computes change in SOC from known organic C-inputs^78^. RothC subdivides the soil into five conceptual SOM pools: decomposable plant material (DPM), resistant plant material (RPM), microbial biomass (BIO), humified organic matter (HUM)) and inert organic matter (IOM). The decay process depends on soil clay content [%], average monthly temperature [°C], precipitation and evapotranspiration [mm], land cover and management, soil depth [cm] and annual C inputs [Mg C ha^−1^] from residues and/or exogenous organic matter (e.g. manure). C-inputs specific to each pool (except for IOM) are described by a rate constant parametrised for grassland, crop and forest land.

Model estimations of SOC turnover, be it produced by Earth system models or by soil/agroecosystem models, and especially at large (e.g. global) scales, are often in disagreement. The causes are multiple, and relate in the case of Earth system models to differences in simulated state variables^79^, and in the case of soil models and agroecosystem models to structural model differences (e.g. humidity and temperature effects sub-models)^80^. For illustration purposes, we retained the RothC model, previously used for global estimations, given its flexibility and suitability under varying pedoclimatic conditions^4,81–83^.

To run RothC for a combination of multiple sites and biopumps we used the SoilR package v1.1^84^, which provides a library of functions and tools under the R environment. For the initialisation of the conceptual carbon pools we used pedotransfer functions (equation and constants provided in Supplementary Methods), which seemed appropriate, as other approaches (e.g. physico-chemical or model equilibrium analysis) are not measurable at the regional scale. Monthly temperature and precipitation data were retrieved from CHELSA^64^, and monthly evapotranspiration from CGIAR’s High-Resolution Global Soil–Water Balance^85^.

The SOC sequestration estimations were complemented with the method described in Lugato et al.^86^ to compute SOC erosion from soil erosion by water (detailed in Supplementary Methods). Input layers for soil erosion were used from the Global Soil Loss map at a 25 km (810 arcsec) resolution for the year 2012^87^, based on a Revised Universal Soil Loss Equation (RUSLE)-based method^88^. Retained cover-management factors required by the SOC erosion method^86^ are listed in Supplementary Methods, Table S9.

The p-values were computed via a paired sample t-test.

## Supplementary Information

Supplementary Methods.

Supplementary Results.

## Data Availability

Dataset required for the framework implementation in Ref.^43^ (https://doi.org/10.48531/JBRU.CALMIP/A3CIFZ).
