# Supplementary material for: A generalizable framework for spatially explicit exploration of soil organic carbon sequestration on global marginal land
Source: Sci Rep. 2022 Jul 1;12:11144. doi: 10.1038/s41598-022-14759-w (PMC9249906; doi:10.1038/s41598-022-14759-w)
Supplement: Supplementary file 1 — Supplementary Methods. [file 41598_2022_14759_MOESM1_ESM.pdf]

Supplementary Methods for:
A generalizable framework for spatially explicit exploration of soil organic carbon sequestration on global marginal land

Ariane Albers[1,*], Angel Avadí[2,3], Lorie Hamelin[1]

[1] TBI, Université de Toulouse, CNRS, INRAE, INSA, Toulouse, France
[2] CIRAD, UPR Recyclage et risque, F-34398 Montpellier, France
[3] Univ Montpellier, CIRAD, Montpellier, France
*Corresponding author: albers.ariane@gmail.com

Contents

Data sources 2
Definition of marginal land and target areas 4
Harmonisation of climate zones 13
Biopump selection and ranking 14
Selection of an adapted soil carbon model 16
RothC initialisation 25
SOC erosion 25
References 26

List of tables

Table S1. List of data sources. 2
Table S2. Marginal land definitions in the literature. 6
Table S3. Biophysical constraints retained by key marginal land mapping studies. 10
Table S4. Land cover classes defined in the FAO Land Cover Classification System (LCCS3). 12
Table S5. World regions, as defined in the CIA Factbook and implemented 12
Table S6. Harmonisation of global climate zone classification systems. 13
Table S7. Criteria for ranking biopumps. 15
Table S8. Main characteristics of soil organic matter models and parameters of for carbon input and mineralisation. 18
Table S9. Mean cover-management factors (C-factors) [dimensionless] per non-arable land-cover types and crop types at European and global scales 25

Data sources

Table S1. List of data sources.

Data	Type	Spatial resolution	Reference	Version/ year	Link
Georeferenced					
World administrative areas (country and sub-national boundaries)	Vector	N/A	Global administrative areas (GADM) maps and data [1]	GADM v3.6, 2018	https://gadm.org/download_world.html
World Regions layer package	Vector	N/A	Esri ArcGIS Data & Maps (2020)	2013	https://www.arcgis.com/home/item.html?id=a79a3e4dc55343b08543b1b6133bfb90
Latitudes and longitude grids	Vector	N/A	Esri ArcGIS Data & Maps (2020)	2014	https://www.arcgis.com/home/item.html?id=ece08608f53949a4a4ee827fd5c30da1
Global Soil Organic Carbon Map	Raster	1 km	FAO GSOC [2]	GSOC v1.5	http://54.229.242.119/GSOCmap/
Global Land Cover Map	Raster	300 m	European Space Agency Climate Change Initiative (ESA-CCI) products [3], based on FAO's Land Cover Classification System v.3 (LCCS3) [4]	2010 and 2018	https://cds.climate.copernicus.eu/cdsapp#!/dataset/satellite-land-cover?tab=form
Global protected areas	Vector	N/A	UN Environment Programme World Conservation Monitoring Centre [5]	WDPA v1.6	https://www.protectedplanet.net/en
Soil and terrain properties	Raster	1 km	Harmonized World Soil Database [6]	HWSD v1.21 (2013)	http://www.fao.org/geonetwork/srv/en/main.home
Global elevation	Raster	1 km	USGS EROS Global 30 Arc-Second Elevation [7]	GTOP030 (1996)	https://www.usgs.gov/centers/eros/science/usgs-eros-archive-digital-elevation-global-30-arc-second-elevation-gtopo30?qt-science_center_objects=0#qt-science_center_objects
Global slope	Raster	1 km	IIASA/FAO Global AgroEcological Zones (GAEZ)	GAEZ v3.0 (2012)	http://www.iiasa.ac.at/Research/LUC/luc07/External-World-soil-database/HTML/global-terrain-slope-download.html?sb=7
Near present (historic) climate	Raster	1 km	Climatologies at High resolution for the Earth's Land Surface Areas [8,9]	CHELSA v1.2, 1979 to 2013)	https://chelsa-climate.org/downloads/
Global climate zones	Vector	N/A	FAO's Global Ecological Zones (GEZ) [10]	GEZ 2010 product, 2nd edition	http://www.fao.org/geonetwork/srv/en/metadata.show?currTab=simple&id=47105
Global soil erosion	Raster	25 km	Global soil loss map [11]	GloSEM v1.1	https://esdac.jrc.ec.europa.eu/content/global-soil-erosion
Actual evapotranspiration	Raster	1 km	CGIAR's High-Resolution Global Soil-Water Balance [12]	2019	https://cgiarcsi.community/data/global-high-resolution-soil-water-balance/

Non-georeferenced					
Key climate and soil requirements of crops	N/A	N/A	FAO Crop Ecological Requirements (ECOCROP) database [13]	2018	https://github.com/supersistence/EcoCrop-ScrapeR
Yield	N/A	N/A	Crops: FAOSTAT [14], lignocellulosic plants [15], grasses (literature)	2010-2018	http://www.fao.org/faostat/en/#data/QC

Definition of marginal land and target areas

Defining land as "marginal" has proved to be challenging [16–18], with some authors even designating it as a non-viable concept [19]. Originally, the concept related exclusively to the economic agricultural framework [20], concerning the reduced productive capacity and benefit for a given land use, often linked with rural poverty [21]. The concept further evolved across disciplines and scales [22], adding biophysical (nature-influenced) and environmental (human-influenced) constraints [23–25], and thus comprising wide-ranging land types: idle, underutilised, unused, barren, inaccessible, degraded, abandoned, fallow or set-aside, wasted, and potentially contaminated (e.g. brownfields, landfills) or reclaimed (e.g. remediated mine land) [17–19,26,27]. Yet, it has been criticised that the umbrella term ignores the criticality as a means of subsistence for marginalised communities, small-scale farmers or indigenous people and the resource and infrastructure requirements for its exploitation [19].

A variety of definitions have been proposed (Table S2).

According to Mellor et al. [18] "the most pronounced problem is related to the variation and ambiguity in its definition or understanding", which has consequently led to methodological inconsistencies. **Agricultural** (potentially suitable for food production historically, currently or in future) and **non-agricultural** (unsuitable/unfavourable for food production) land types include the following classifications [18]:

- Agricultural land type comprises areas that can potentially become productive, despite current biophysical constraints (e.g. sandy, acid or saline soils, highly erodible, or soils prone to droughts, compaction, floods, and sloppy terrains). It covers **degraded** (reduced soil fertility and productivity), fallow (temporary suspension as a crop rotation period), **abandoned** (due to declining yields), **reclaimed** (from previously unsuitable conditions) **and wasted** (active dunes, salt flats, rocky outcrops, deserts, ice caps and arid mountain regions) land.
- Non-agricultural land type refers to **mine land** (abandoned after mineral exploitation), brownfields (previously used but currently not fully used), **landfills** (waste disposal sites) as well as **buffers** (including utilities and urban land such as parks, roadsides).
- Both land types represent **contaminated land** (e.g. with metals, petroleum, aromatic and chlorinated hydrocarbon, organic compounds), which can potentially be used after remediation (e.g. phytoremediation) or restoration and under consideration of safety and environmental measures within the contaminated and surrounded areas.

Degraded land, as recently defined in the IPCC [28] refers to as "a negative trend in land condition, caused by direct or indirect human-induced processes, including anthropogenic climate change, expressed as long-term reduction or loss of at least one of the following: biological productivity, ecological integrity or value to humans"

Key marginal land mapping studies have retained slightly different sets of biophysical criteria to identify and map marginal lands (

Table S3). Elbersen et al. [17] identified a set of biophysical, land use management, socio-economic and ecosystem services constraints to map marginal land suitable for industrial crops in Europe in the context of the EU H2020 MAGIC project. The biophysical (i.e. natural) criteria were retained, following an approach by the Joint Research Centre [29]: adverse climate (low temperature, dryness), excessive wetness (excess soil moisture, limited soil drainage), adverse chemical composition (salinity, sodicity, natural toxicity, toxicity by pollutants), low soil fertility (pH, SOC), limitations in rooting (unfavourable soil texture, coarse fragments, organic soils, surface rockiness, shallow rooting depth), adverse terrain conditions (steep slope, flooding risk). An assessment of biomass resources from marginal lands in Asia-Pacific Economic Cooperation economies [24] retained terrain (slope) constraints and soil problems. The latter are roughly equivalent to

MAGIC's "limitations in rooting" group of constraints and FAO's classification of problem soils/degraded lands [30].

A key component of marginal lands is **abandoned agricultural land**, which in our definition (see main article) corresponds to recent conversion of agricultural land to mosaic cropland/natural vegetation (complemented with mosaic cropland/natural vegetation to semi-natural), grasslands, sparse vegetation, bare areas, mosaic herbaceous cover or shrubland. Land cover classes corresponding to FAO Land Cover Classification System (LCCS3) [4] are listed in Table S4.

To define target areas, as discussed in the main article, all marginal lands within the same GEZ and geo-political world region (listed in) were consolidated and their values averaged, as previously done for global assessments requiring characterisation of larger regions with data at a finer granularity (e.g. [31]).

Table S2. Marginal land definitions in the literature.

Term used	General Definition	Synonyms/ land use characterisations	Scale	Crops	GIS	Sour-ce
Marginal land	(a) abandoned agricultural land and set aside for conservation purposes, b) buffer strips along rivers and streams or riparian buffers, c) buffer strips along roads or roadway buffers,d) brownfield sites that have been contaminated as a result of past practices.	Fallow and idle cropland, grass- and pasture land herbaceous wetlands	NE, USA, regional	cellulosic biofuels	x	[32]
Marginal land	Poor climate, poor physical characteristics, or difficult cultivation. Limited rainfall, extreme temperatures, low quality soil, steep terrain, or other problems for agriculture.	Bare and herbaceous areas; intensive and extensive pastoralism; moderate to steep slope; lands with soil problems, deserts, high mountains, land affected by salinity, waterlogged or marshy land, barren rocky, and glacial areas.	APEC		x	[24]
Agricultural marginal land	Currently abandoned marginal land or set-aside		Italia, local	Poplar, Robinia, willow, sorghum	x	[33]
Marginal rent	The poorest lands utilized above the margin of rent-paying land with respect to the next lower purpose.					[34]
Marginal land	Limitations which in aggregate are severe for sustained application of a given use. Increased inputs to maintain productivity or benefits will be only marginally justified. Limited options for diversification without the use of inputs. With inappropriate management, risks of irreversible degradation.					[21]
Marginal land	Depends on the interaction of physical, environmental, social and economic aspects. Implies that abandonment can occur everywhere, even in areas with a high yield potential, and even in a satisfying general economic situation.	Set-aside, abandonment. Land uses that are at the margin of economic viability.				[35]
Marginal land	Limited productive or regulatory function	Degraded land				[36]
Abandoned agricultural lands	Land that have been abandoned to crop and pasture due to the relocation of agriculture and due to degradation from intensive use.	Agriculturally degraded land.<br>Crop and pasture land transitions to other land uses, expect of crop to pasture, pasture to crop, agriculture to forest, and agriculture to urban.	Global		x	[37]
Abandoned agricultural land	Soils of abandoned areas are generally of low quality and thereby limited suitability for crop production.		Estonia, regional			[38]

Term used	General Definition	Synonyms/ land use characterisations	Scale	Crops	GIS	Sour-ce
Marginal agricultural land	Soils low inherent productivity for agriculture, is susceptible to degradation, and is high-risk for agricultural production.	abandoned farmland, degraded land, wasteland, and idle land	Semi-Global (Africa, China, EU, India, South America, US)		x	[39]
Degraded and marginal land	Limited usefulness for any production or regulation function	Degraded, unproductive, low-productive, idle, wasted, fallow				[40]
Marginal agricultural land	N/A	May be characterized by degraded soils, particularly saline soils.	Australia			[41]
Marginal land	Not currently used for crop	Idle, biophysically marginal	USA			[42]
Surplus land	Area where cost-effective production, under given environmental conditions, cultivation techniques, agriculture policies as well as macro-economic and legal conditions is not possible.	Fallow land, set-aside, abandoned land, degraded land, marginal land (idle, under-utilised, barren, inaccessible). Exclude agriculture or forestry for reasons other than poor availability of natural resources (e.g. socio-economic or political reasons).	Global	Industrial crops		[43]
Agricultural marginal or set-aside land	Comprises all non-cultivated areas where actual primary production is too low to allow competitive agriculture, whereas degraded land refers to land previously cultivated and now marginal, due to soil degradation or other impacts resulting from inappropriate management or external factors.	Idle, degraded, under-utilized lands, wastelands and abandoned croplands	Italy, regional	Brassica	x	[44]
Marginal agricultural land	Not profitable for food crops due to low productivity.	Shrubland, grassland	Canada	Switchgrass, poplar	x	[45]
Marginal land	Relatively poor natural condition but is able grow energy plants, or land that currently is not used for agricultural production but can grow certain plants.	Woodland (shrub land, sparse forest land), grassland and barren land (including shoal/bottomland, saline and alkaline land, and bare land). Shrub, high/moderate grassland cover excluded due to eco-environmental security.	China, regional	Cassava-bioethanol	x	[45]
Marginal land	Unsuitable for crop production, but ideal for the growth of energy plants with high stress resistance. These lands include barren mountains, barren lands and alkaline lands	Shrub land, Sparse forest land, dense grassland, moderate dense grassland, sparse grassland, shoal/bottomland, alkaline land, bare land	China, regional	Pistacia chinensis biodiesel	x	[46]

Term used	General Definition	Synonyms/ land use characterisations	Scale	Crops	GIS	Sour-ce
Marginal lands	• Physically: unsuitable for any form of land management or agricultural production (e.g. rocky land with little soil, flooding or ponding areas)<br>• Biologically: biological stresses and fragile or harsh natural conditions (e.g. coldness, drought, high or low pH soils).<br>• Environmentally: high risks or damages of environmental and ecological functions (e.g. areas of high biodiversity, wetlands).<br>• Economically: not profitable regarding the cost-benefit of production	Abandoned, degraded, fallow, wasteland, unused, idle.<br>Any other land not specifically listed under: arable land and land under permanent crops, permanent pastures, forests and woodland, built on areas, roads or barren lands				[19]
Marginal land	1) not fit for food production, 2) ambiguous lower quality land, 3) economically marginal land	set-aside, idle, unused, suitable, free, spare, abandoned, under-used, set aside, degraded, fallow, additional, appropriate, under-utilised.				[47]
Urban marginal lands	Lots and pastures characterized by poor agricultural potential, ill-suited for residential purposes, and otherwise economically unprofitable.	Vacant and abundant lands. Include urban commercial lands:<br>Strip mines, Gullied land, Gravel pits, Quarries, Coal dump, Industrial dump, Slope less than 15%	Pittsburgh, USA, local	Sunflower biofuel	x	[48]
Marginal land	Typically characterized by low productivity and reduced economic return or by severe limitations for agricultural use. Land can be marginal physically, biologically, environmentally-ecologically, economically.	Fragile, unproductive lands, waste lands, under-utilized lands, idle lands, abandoned lands, or degraded lands.	USA, regional	Lignocellulosic biomass crops		[22]
Marginal land (non-arable)	Poorly suited for food crops because of low productivity due to inherent edaphic or climatic limitations or because they are located in areas that are vulnerable to erosion or other environmental risks when cultivated.		USA, regional	Alfalfa, poplar, corn, soybean, wheat	x	[23]
Marginal land	Areas with inherent disadvantages or lands that have been marginalized by natural and/or artificial forces. These lands are generally underused, difficult to cultivate, have low economic value, and varied developmental potential.	Abandoned, disturbed underutilised, wasted, limbo, degraded<br>Idle, abandoned cropland, barren lands, transmission lines, roads, rails, abandoned minelands, landfills.	USA, regional	Renewable energy technologies	x	[49]
Urban marginal lands	Not suitable for primary agriculture, has a soil slope <15% and has a minimum parcel size.	Private marginal vacant lands.<br>Excluded saline lands, abandoned or degraded forests.	Boston, USA (spatial)	Miscanthus, poplar, willow	x	[50]

Term used	General Definition	Synonyms/ land use characterisations	Scale	Crops	GIS	Sour-ce
Degraded land	Nearly universal consensus that degradation can be defined as a reduction in productivity of the land or soil due to human activity.	Degraded (encompassing desertification, salinization, erosion, compaction, or encroachment of invasive species, overutilization, etc, marginal land, abandoned cropland	Global		x	[27]
Marginal land	Chinese classification system defines: shrub land, sparse forest land, sparse grassland, shoal, bottomland, sand land Gobi Desert, alkaline land, wetland, bare and bare rock land.	This study excluded: Shrub land, Sparse forest<br>Gobi Desert, Wetland	China	Miscanthus	x	[51]
Marginal land	Determined with respect to the particular economic opportunities offered by land-use choices	Economically marginal land classified into this “natural” land category (includes “rewilded” areas).				[52]
Marginal land or degraded lands	Soils that have physical and chemical problems or are uncultivated or adversely affected by climatic conditions.	highly erodible, flood-prone, compacted, saline, acid, contaminated, or sandy soils, reclaimed minesoils, urban marginal sites, and abandoned or degraded croplands	-	black locust, poplar, willow		[25]
Marginal land	Lands with poor soil quality and weak agricultural yield potentials. Four clusters: 1) post-mining sites, 2) abandoned former arable land, 3) post-industrial site (railway), and 4) already marginal due to poor soil conditions.	fallow, set-aside, abandoned arable, anthropogenically degraded, or waste land, mountainous	EU	Black locust, black pine; basket willow, poplar, miscanthus, switchgrass	x	[53]
Marginal and degraded land	Specific land use types, with marginal soil quality and flat to moderate soil slopes		101 cities (around Boston)	Miscanthus, willow, poplar, switchgrass		[54]
Marginal land	Low production, also with limitations that might make them unsuitable for agricultural practices and important ecosystem functions.		EU			[55]
Marginal land	Lands having limitations which in aggregate are severe for sustained application of a given use and/or are sensitive to land degradation, as a result of inappropriate human intervention, and/or have lost already part or all of their productive capacity as a result of inappropriate human intervention and also include contaminated and potentially contaminated sites that form a potential risk to humans, water, ecosystems, or other receptors.	areas with natural constraints, fragile, degraded, contaminated and potentially contaminated lands	EU			[17]

Term used	General Definition	Synonyms/ land use characterisations	Scale	Crops	GIS	Sour-ce
Marginal land	Any identifiable land area, whether originally agricultural or non-agricultural, including those in urban areas, which is currently unused or underutilised due to economic, environmental or social factors, but which is suitable for temporary or longer-term use for sustainable energy production.	Fallow or set-aside, abandoned (farmland), wasted, degraded, brownfields, reclaimed				[18]

Table S3. Biophysical constraints retained by key marginal land mapping studies.

Constraint category	A. FAO (agricultural problem-land approach) [30]	B. JRC[29] and MAGIC [17]	C. APEC [24]	Data sources
Adverse climate				
Low temperature	Polar/boreal	LGP ≤180 days	N/A	A: GAEZ/FAO problem lands [56]
Dryness	LGP ≤60 days	Severe: P/PET ≤ 0.5<br>Sub-severe: P/PET ≤ 0.6	N/A	A: GAEZ/FAO problem lands (warning) [56]
Excessive wetness				
Excess soil moisture	Waterlogged and/or flooded for a significant part of the year	Severe: 210 days at or above FC<br>Sub-severe: 190 days at or above FC	Poorly and imperfectly drained soils	C: HWSD [6]
Limited soil drainage		High water table throughout the year: wet 80 cm > 6 months, or 40 cm > 11 months		
Adverse chemical conditions				
Salinity	Saline/sodic	dS/m >15	Salt-affected soils: Solonchaks, Solonetz, and Solodic Planosols	B: HWSD [6]
Sodicity		ESP ≥15%		B: HWSD [6]
Natural toxicity / acid soils	Accumulation of sulphitic materials under brackish water	High content of sulphur that have acidification potential upon drainage	Severe: pH <4.5<br>Sub-severe: 4.5 > pH > 5.5	B: HWSD [6]
Low soil fertility				
Soil reaction		pH <4.5 or >8	pH <5.5<br>Calcisols<br>Gypsic horizon	B: HWSD [6]
Fertility	Infertile (severe nutrient deficiency)	Severe: SOC in top soil (30 cm) <0.5%	Low to moderate natural fertility	B: HWSD [6] and GSOC [57] (<30 t C/ha, following the SOC_{stock} equation in [58]

		Sub-severe: SOC in top soil (30 cm) <0.75%		A: GAEZ/FAO problem lands (warning) [56]
Limitations in rooting				
Unfavourable soil texture	<18% clay and >65% sand; heavy cracking clays (Vertisoils)	Severe: >70% sand<br>Sub-severe: >60% sand	Heavy cracking clays (Vertisoils)	B: HWSD [6]
Coarse fragments and surface stones	Rocky	>35% coarse fragments and/or >15% rocks of topsoil	Arenosols, Regosols, and Vitric Andosols with coarse texture; soils with petric and stony phase	A: GAEZ/FAO problem lands (warning) [56]
Organic soils	Peat >40 cm	>30% organic matter	Peat soils (Histosoils)	B: HWSD [6]
Shallow rooting depth	<50 cm	<30 cm	<50 cm	A: GAEZ/FAO problem lands
Adverse terrain conditions				
Slope	Dominant slope >30%	Severe: >80% area has slope >15%<br>Sub-severe: >60% area has slope >15%	Severe: 16-30%<br>Sub-severe: 8-16%	A: GAEZ/FAO problem lands [56]
Flooding risk	Waterlogged and/or flooded for a significant part of the year<br>Alluvial soil in deserts	Severe: >2 m flood in 2 years<br>Sub-severe: 1-2 m flood in 2 years	N/A	A: GAEZ/FAO problem lands (warning) [56]

Notes. LGP: Length of Growing Period. P: precipitation. PET: potential evapotranspiration. FC: field capacity. ESP: saturation with exchangeable sodium. dS: deciSiemens.

Table S4. Land cover classes defined in the FAO Land Cover Classification System (LCCS3).

Land cover class	LCCS3 code
Cropland, rainfed	10
Cropland, irrigated or post flooding	20
Mosaic cropland (>50%) / natural vegetation (tree, shrub, herbaceous cover) (<50%)	30
Mosaic natural vegetation (tree, shrub, herbaceous cover) (>50%) / cropland (<50%)	40
Tree cover, broadleaved, evergreen, closed to open (>15%)	50
Tree cover, broadleaved, deciduous, closed to open deciduous, closed to open (>15%)	60
Tree cover, needle leaved, evergreen, closed to open (>15%)	70
Tree cover, needle leaved, deciduous, closed to open (>15%)	80
Tree cover, mixed leaf type (broadleaved and needle leaved)	90
Mosaic tree and shrub (>50%) / herbaceous cover (<50%)	100
Mosaic herbaceous cover (>50%) / tree and shrub (<50%)	110
Shrubland	120
Grassland	130
Lichens and mosses	140
Sparse vegetation (tree, shrub, herbaceous cover) (<15%)	150
Tree cover, flooded, fresh or brackish water	160
Tree cover, flooded, saline water	170
Shrub or herbaceous cover, flooded, fresh/saline/brackish water	180
Urban areas	190
Bare areas	200
Water bodies	210
Permanent snow and ice	220

Table S5. World regions, as defined in the CIA Factbook and implemented

World region	Esri[a] code
Antarctica	1
Asiatic Russia	2
Australia/New Zealand	3
Caribbean	4
Central America	5
Central Asia	6
Eastern Africa	7
Eastern Asia	8
Eastern Europe	9
European Russia	10
Melanesia	11
Micronesia	12
Middle Africa	13
Northern Africa	14
Northern America	15
Northern Europe	16
Polynesia	17
South America	18
Southeastern Asia	19
Southern Africa	20
Southern Asia	21

Southern Europe	22
Western Africa	23
Western Asia	24
Western Europe	25

[a] https://www.arcgis.com/home/item.html?id=84dbc97915244e35808e87a881133d09

Harmonisation of climate zones

Adaptation of climate zone classes used in ECOCROP [13] based on the Köppen climate classification [59] with Global Ecological Zoning (GEZ) framework [10], updated for 2010, considering the separate classification of "mountain system" (see definition) in GEZ based on Köppen-Trewartha [59,60], due to high variations of both vegetation formations and climatic conditions [10].

Criteria described in [10] (Table 7, p. 15). See also http://www.fao.org/3/ad652e/ad652e07.htm#P796_39239 and http://www.fao.org/3/ad652e/ad652e17.htm. Table S6. shows the harmonisation of climate between both classification systems.

Table S6. Harmonisation of global climate zone classification systems.

Code	FAO GEZ	ECOCROP
gez1	Boreal coniferous forest	Boreal
gez2	Boreal mountain system	Boreal
gez3	Boreal tundra woodland	Boreal
gez4	Polar	N/A
gez5	Subtropical desert	Desert or arid
gez6	Subtropical dry forest	Subtropical dry summer
gez7	Subtropical humid forest	Subtropical humid
gez8	Subtropical mountain system	Subtropical dry summer & Subtropical dry winter
gez9	Subtropical steppe	Steppe or semi-arid
gez10	Temperate continental forest	Temperate continental
gez11	Temperate desert	Desert or arid
gez12	Temperate mountain system	Temperate humid winter & Temperate dry winter
gez13	Temperate oceanic forest	Temperate oceanic
gez14	Temperate steppe	Steppe or semi-arid
gez15	Tropical desert	Desert or arid
gez16	Tropical dry forest	Tropical wet & dry
gez17	Tropical moist forest	Tropical wet & dry
gez18	Tropical mountain system	Tropical wet & dry
gez19	Tropical rainforest	Tropical wet
gez20	Tropical shrubland	Steppe or semi-arid
gez21	Water	N/A

Definition of Ecological Zone [10] (p.10): "zone or area with broad yet relatively homogeneous natural vegetation formations, similar (not necessarily identical) in physiognomy. Boundaries of the Ecological Zones approximately coincide with Köppen-Trewartha climatic types, which are based on temperature and rainfall. An exception to this definition are "mountain systems", classified as one separate Ecological Zone in each domain and characterized by a high variation in both vegetation formations and climatic conditions".

Biopump selection and ranking

The pre-selection of potential biopumps was based on a semi-quantitative analysis. Table S7 shows the criteria considered for the scoring and ranking procedure and main sources of data and information.

The first criterion quantified two main attributes considering annual SOC stock changes [t C ha^{-1} yr^{-1}] and belowground C input fraction [t C ha^{-1}]. SOC changes were computed from [58] considering land transformation from fallow, short-rotation coppice, crop-, grass-, and forest land to perennial crops; for both top- (≤30 cm) and sub- (>30 cm) soils per tropical, subtropical and temperate climate zones (here the reported boreal zone was linked to temperate and the arid and Mediterranean zones to subtropical zones). Belowground root C allocation was based on the plant fractioning and carbon partitioning approach [61] calculated from the leaf, stem and root mass fractions [g g^{-1}], yield data [t ha^{-1}], harvest index [%] [62,63] and belowground C content [%] per crop type [64]. It has been suggested that C inputs to the soil may provide a more robust estimate than a fixed shoot:root ratio [65]. Moreover, about half of the C assimilated by plants is transferred to the soil [66].

The second criterion quantified the productivity in terms of mean, min and max yields [t ha^{-1} yr^{-1}] expressed in dry mass [62]. Data for agricultural crops were retrieved from FAOSTAT [14] for the years 2010 to 2018, corresponding to values from all known regions and the global mean. For lignocellulosic crops, data were retrieved from Li et al. [15], mostly experimental data over several consecutive years. For the remaining innovative crops, data were retrieved from various peer-reviewed sources.

The third criterion qualified marginal land suitability [67]. Species with high abiotic stress tolerances (e.g. to droughts, frost, sandy soils, etc.) and other relevant features associated with marginal land (e.g. phytoremediation properties, low input) were scored higher.

We evaluated the biopumps by re-scaling quantitative data, assigning scores, weighting, standardising, and ranking (Table S7). Re-scaling was necessary to obtain a common numerical scale by normalising the values between zero and one [0;1] based on the Min-Max scalar, where the range of the values change but the shape of the data is conserved. The values were then scored in ascending order: very low [0], low [1], moderate [2], good [3], and high [4]. Next, the scores were weighted based on the arithmetic weighted mean followed by a statistical standardisation via the z-score. Finally, values with negative standard deviation (i.e. all scoring below the mean) were excluded, and all positive ones ranked with the best observation close to the maximum.

Table S7. Criteria for ranking biopumps.

			Score	0 - very low	1 - low	2 - moderate	3 - high	4 - very high	
			**Re-scale**	**0-2**	**2-4**	**4-6**	**6-8**	**8-10**	
**Criteria**	**Criteria description**	**Weight**	**Unit**						**Main source**
Annual SOC stock changes	Top- (0-30 cm) and subsoil (x > 30 cm)	30%	t C ha^{-1} y^{-1}			*			[58]
	LUC attributes. Transformation to perennials from previous annual crop, grassland, fallow, and short rotation coppice, natural forest and primary forest.		t C ha^{-1} y^{-1}			*			
	Climate zone attributes: Tropical, Subtropical and Temperate		t C ha^{-1} y^{-1}			*			
Sequestration potentials	Associated to a crop family from literature review	20%	n/a	Oilseed, vegetable, tuber	Fibre	Cereals, legume	Grasses, palm	Woody: orchard, shrub, SRC	[68]
Root C	Belowground C in the living roots or rhizome deposition partitioned to the soil.	25%	t C ha^{-1}			*			Large literature review on yields (e.g. [14]) and allometric relations [15]
"Marginality"	Abiotic stress tolerance to grow on marginal land. Climatic: arid zones, cold climate, resistance to dry climates and extreme temperatures (droughts, heat stress or low temperature and frost), as well as has a high tolerance to excessive wetness. Soil: sandy soils with low SOM; heavy cracking clays (Vertisoils); soils with coarse texture (Arenosols, Regosols, and Vitric Andosols); soils with petric and stony phase, saline/sodic, acid sulphate soils. Other: low-input crops, marginal land properties	15%	n/a	No stress tolerance	climatic tolerance but special soil texture preferences	climatic tolerance OR unfavourable/ poor soil texture and chemical conditions	climatic tolerance AND unfavourable/ poor soil texture and chemical conditions	climatic tolerance AND unfavourable/poor soil texture and chemical conditions AND low input crops OR remediation/phyto -sanitation properties	EU MAGIC project [67,69]
Economic yield	High yield productivity (primary use) can be attractive for bioeconomic supply chains.	10%	t ha^{-1} y^{-1}			*			

* MinMax Scalor

Selection of an adapted soil carbon model

The selection of a model for predicting soil carbon sequestration (SCS) is not straightforward, as no single one clearly outperform the others [70] and multi-model comparisons have not been conclusive on a particular model [71]. The number of models describing biogeochemical processes in the soil has increased considerably since the 1930s to more than 250 distinctive ones [72]. A minor subset of available models is widely used, where the most cited ones are Century, RothC, DNDC, EPIC and DSSAT [71]. Soil models generally differ vis-à-vis model structure (from simple mineralisation to integrating the soil-plant dynamic and multiple flow exchanges), number of conceptual C pools (most comprising 2-5 pools), as well as spatial (from soil aggregates to landscape applications) and temporal (hour to centuries) resolutions. Most models include soil organic matter (SOM) dynamics. The mathematical formalism for SOM decay proposed by Hénin and Dupuis [73] is implemented in most models. It follows a simple first order differential equation with constant rates as a function of time, which is controlled by a variety of external climatic and edaphic factors (e.g. temperature, moisture, pH, texture and clay mineralogy), as well as land use and land management practices [74,75]. A comparison of commonly used SOC models is presented in Table S8.

To choose a model for the proposed framework, we followed the rating criteria presented in Köck et al. [76] for Tier 3 GHG inventory reporting [77] and the technical guidelines for spatially explicit modelling of SCS and mapping by the FAO [78]. An essential criterion is the model capacity to represent carbon dynamics at a wide range of spatial and temporal resolutions, which basically segregates the models into “types” 1 and 2 [79]. The former model SOM dynamics with “no dynamic vegetation component” [72], as the C inputs are based on simple allometric relations [74], which requires less inputs and predicts the net SOC change at lower level of temporal resolutions. The latter belong to the (agro-)ecosystem models, and represent a large phase-space dimension [72] determined by a number of sub-models, parameters and measurements at high temporal resolutions. Our selection focused on **type 1 models**, as a high-level resolution was not deemed necessary for long-term simulations at regional scales.

Further criteria were considered: land use category (at least crop and grassland at different altitudes), soil type (excluding organic soils), soil depth (mainly topsoil), management practices (e.g. external C inputs from fertilisation and amendments). Models fulfilling most of the retained criteria were RothC and C-tool. The overall performance of these models, as compared to that of type 2 ones, has been shown to be good. C-tool showed similar C and N interactions when compared to DAISY [79], while RothC produced similar results as Century [80,81].

The Rothamsted C model, RothC [82,83], computes change in SOM from known C inputs [84]. It uses a monthly time step and subdivides the soil into five conceptual SOM pools: decomposable plant material (DPM), resistant plant material (RPM), microbial biomass (BIO), humified organic matter (HUM)) and inert organic matter (IOM). C inputs are first allocated to DPM (fast turnover) and RPM (slow turnover) based on the DPM:RPM ratio determined by the quality and distribution of plant input throughout one year, yet the distribution is insensitive to long-term C inputs, which makes the model applicable globally [85]. The decay process depends on soil clay content [%], average monthly temperature [°C], precipitation and evapotranspiration [mm], land cover and management, soil depth [cm] and annual C inputs [t C ha^{-1}] from residues and/or exogenous organic matter (e.g. manure). C inputs specific to each pool (except for IOM) are described by a rate constant parametrised for grassland, crop and forest land. RothC has been used in a wide range of climates and regions of the world (more than 80 countries) in combination with GIS products [85–87], and is currently recommended as a standardised spatialised SOC model for national comparisons at a 30 arcsec resolution [78]. The latest version is RothC v26.3 [84], but a series of versions (e.g. RothPC-1 to simulate andosols subsoil C [88,89], RothC10_N for dry soils in arid and semiarid regions [90]) and methods (e.g.

initialisation without historic data for wide ranging soil conditions [91]) have been developed. Main persisting limitations of the model include permanent waterlogged soils and organic soils [90].

Table S8. Main characteristics of soil organic matter models and parameters of for carbon input and mineralisation.

Model	Original location	C pools (residence time in years)	Land use type	Spatial resolution						Temporal resolution				C inputs	Parameters influencing mineralisation	C:N	14C	C output (soil depth in cm)	Download/ documentation URL
				P	F	CT	RG	NA	GL	S	M	L	step						
**Simple, empirical models**																			
IPCC 1-2 Tier (IPCC 2006, Chapter 4)	Global	Dead organic matter (DOM) of wood and litter	Grassland, Cropland, Forest land				x	x	x		x		year	DOM, Default carbon stocks and C change factors; replaced by country-specific values in Tier 2.	Country-specific factor for climate and soil types, and/or land use class in Tier 2.			Annual SOC change (0-0.30)	https://www.ipcc-nggip.iges.or.jp/public/2006gl/vol4.html
Hénin-Dupuis model [73]	France	Fresh organic matter Active pool						x			x	x	year	Residue, manure, initial SOC				Annual SOC change (0-0.30)	
**Soil process models**																			
AMG [93,94], AMGv.2 [95]	France	Fresh organic matter Active carbon (3.5) Stable carbon	Cropland	x	x			x				x	year	yield allometric relation, manure, initial SOC	Temp., Precip., EVT, soil tillage depth, irrigation, clay and carbonates, pH, BD	x		Annual SOC change (0-0.30)	https://www6.hautsdefrance.inrae.fr/agroimpact/Nos-dispositifs-outils/Modeles-et-outils-d-aide-a-la-decision/AMG-et-SIMEOS-AMG/AMG-model-description
RothC (Rothamsted carbon model) [83],	UK	Decomposable plant material (0.1) Microbial biomass (1.5)	Grassland Cropland Forest land	x	x	x	x	x	x		x	x	month	residue, roots, manure, initial SOC	Temp., Precip., EVT, water, soil cover, soil depth, clay, DPM:RPM ratio	x	x	Annual SOC change (0-0.30), microbial biomass C,	https://www.rothamsted.ac.uk/rothamsted-carbon-model-rothc

Model	Original location	C pools (residence time in years)	Land use type	Spatial resolution						Temporal resolution				C inputs	Parameters influencing mineralisation	C:N	14C	C output (soil depth in cm)	Download/ documentation URL
				**P**	**F**	**CT**	**RG**	**NA**	**GL**	**S**	**M**	**L**	**step**						
		Resistant plant material (3.3)<br>Humus (50)<br>Inert organic matter (50 000)																change in 14C	
ICBM (Introductory carbon balance model) [96,97]	Sweden	Young (1.25)<br>Old (166)<br>Inert (can be added)	Cropland in cool temperate climate, Grassland				x	x	x		x		year<br>day	yield allometric relation, manure	Temp., water, cultivation			Annual SOC change (0-0.25)	
C-TOOL [98,99] CN-SIM for N dynamic [100]	Den-mark	Fresh organic matter (0.6-0.7)<br>Active Humus (50)<br>Resilient organic matter (600-800)	Cropland	x			x	x				x	month	yield allometric relation, manure	Temp., clay, BD, initial SOC	x	x	Annual SOC change (0-25 and 25-100)	https://pure.au.dk/portal/en/publications/id(ec9459f6-e147-4ac5-ae9f-dd5670ee514f).html
NCSOIL (Nitrogen and carbon transformation in soil) [101]		Residue pool<br>Pool I labile (0.01)<br>Pool I resistant (0.07)<br>Pool II labile (0.45)<br>Pool II resistant (1.72)<br>Pool III (stable humus) (25.0)	Cropland<br>Grassland<br>Forest land							x			day		Temp., clay, N, water	x		C and N flows	

Model	Original location	C pools (residence time in years)	Land use type	Spatial resolution									Temporal resolution	C inputs	Parameters influencing mineralisation	C:N	14C	C output (soil depth in cm)	Download/ documentation URL
				P	F	CT	RG	NA	GL	S	M	L	step						
Yasso [102], Yasso15 [103]	Finland	4 Chemically distinguishable fractions of fresh OM: Ethanol soluble (E), Water soluble (W), Acid soluble (A), Non-soluble (N), and 1 Humus fraction	Forest land	x	x		x	x	x	x	x	x	month, year	Litter (quantity, quality and diameter)	Temp., Precip.	x		Annual Soil C change (0-100)	https://en.ilmatieteenlaitos.fi/yasso-download-and-support
SOMM (Soil organic matter mineralization) [104]		Undecomposed litter, Litter impregnated by humic substance, humic substances of mineral top soil	Natural vegetation grassland, forest land	x	x							x	day year	Litter	substrate factors from microbial species, N and ash content			Soil C change (upper soil layer)	
SOCRATES [105]		Decomposable plant material (0.02) Resistant plant material (0.32) Unprotected MB (0.003) Protected MB (0.35)	Grassland Cropland	x									week, year	NPP partitioning, initial SOC	Temp., Precip., clay, cation exchange capacity, BD			Change in Soil C	

Model	Original location	C pools (residence time in years)	Land use type	Spatial resolution									Temporal resolution	C inputs	Parameters influencing mineralisation	C:N	14C	C output (soil depth in cm)	Download/ documentation URL
				P	F	CT	RG	NA	GL	S	M	L	step						
		Stable OM (21.31)																	
**(Agro-) Ecosystem models (involving several sub-models: modules e.g. plant-growth, soil-water balance, etc.)**																			
CENTURY [106,107]	USA	Active SOM (0.5-1)<br>Slow SOM (10-50)<br>Passive SOM (400-4000)	grassland, cropland forest land, natural vegetation, Savannah	x	x		x	x	x			x	month	From simulated plant production, fertiliser, initial SOC	Min and max Temp., Precip., lignin content, plant and soil N, P, and S content, soil texture (sand, clay, silt fractions), pH, BD, irrigation, crop sequence, grazing, etc.	x	x	C and N dynamic or C, N and P dynamic or C, N, P and S dynamic (0-0.20)	https://www2.nrel.colostate.edu/projects/century5/; https://www2.nrel.colostate.edu/projects/century/MANUAL/html_manual/man96.html#OUT_VARS
ECOSSE (Estimation of Carbon in Organic Soils – Sequestration and Emissions) [108]	UK	Humus<br>Biomass<br>Resistant plant material<br>Decomposable plant material<br>Inert pool	Cropland (mineral and organic soils)		x			x					month year	Plant growth, fertiliser, Initial SOC,	Temp., water, DPM:RPM ratio, soil cover, Soil characteristic for each soil horizon, content of C, clay, pH, silt and sand, bulk density, timing of management	x		C and N dynamics (0.05-300), and GHG emissions	
DAYCENT [109–111]	USA	Active SOM (1-5)<br>Slow SOM (10-50)<br>Passive SOM (400-2000)	Cropland	x	x								day	Fertiliser, initial SOC, initial N, P, S	Temp., Precip., soil texture (sand, clay, silt), BD, irrigation, crop cover, crop sequences and timing management			Includes NO_2 emissions	https://www2.nrel.colostate.edu/projects/daycent-downloads.html
DNDC (DeNitrification DeComposition) [112]	USA	Very labile litter (0.04)<br>Labile litter (0.04)<br>Resistant litter (0.14)<br>Labile microbial	Cropland Wetland	x	x	x	x		x	x	x	x	Day to year	Plant growth, fertiliser	Temp., water, N, clay, tillage	x		C dynamic, nitrogen leaching, nitrous oxide (N2O), nitric oxide (NO), dinitrogen	https://www.dndc.sr.unh.edu/

Model	Original location	C pools (residence time in years)	Land use type	Spatial resolution						Temporal resolution				C inputs	Parameters influencing mineralisation	C:N	14C	C output (soil depth in cm)	Download/ documentation URL
				**P**	**F**	**CT**	**RG**	**NA**	**GL**	**S**	**M**	**L**	**step**						
		biomass (0.01) Resistant microbial mass (0.07) Labile humads (0.02) Resistant humads (0.45) Passive humads																(N2), ammonia (NH3), methane (CH4) and carbon dioxide (CO2) flows from fermentation, denitrification and nitrification sub-models	
EPIC (Environmental Policy Integrated Climate – soil erosion calculator [113]	USA	Fresh pool (33) Active pool Stable pool	Cropland	x	x		x	x		x	x	x	Day	Plant growth, fertiliser,	Temp., water, N, clay, crop cover, tillage, crop rotations, cation exchange capacity	x		water balance, sediment, fate and transport of sediment/N/P/C and chemicals, net ecosystem exchange, cost of erosion	https://epicapex.tamu.edu/epic/
DAISY [114]	Den-mark	Added OM 1 (slow - 0.06) Added OM 2 (fast - 0.04) Soil microbial biomass 1 (slow- 0.28)	Cropland in cool temperate climate	x	x	x				x	x	x	Hour and day	Plant growth, fertiliser	Temp., Precip., EVT, global radiation, Soil texture, humus, tillage, irrigation, sowing, harvesting	x		Change in Soil (C) quality, production, and leaching impacts	https://daisy.ku.dk/

Model	Original location	C pools (residence time in years)	Land use type	Spatial resolution						Temporal resolution				C inputs	Parameters influencing mineralisation	C:N	14C	C output (soil depth in cm)	Download/ documentation URL
				P	F	CT	RG	NA	GL	S	M	L	step						
		Soil microbial biomass 2 (fast- 2.78)<br>SOM 1 (slow-1000)<br>SOM 2 (fast-20)																	
CANDY (Carbon and nitrogen dynamics) (Franko et al. 2002)	Ger-many	Fresh organic matter (2.5)<br>Biologically active SOM (7.14)<br>Slow cycling SOM (20)<br>Inert SOM	Cropland in cool temperate climate	x	x	x	x	x		x		x	Day, year	Plant growth, initial SOC, fertiliser	Temp., Precip., N, radiation; moisture, clay, silt , BD, tillage, irrigation	x		C and N dynamics (0.10-200), water balance, Crop N uptake	https://www.ufz.de/index.php?en=39725
PaSim (Pasture Simulation model) [117] Thornley et al., 1998; Riedo et al., 1998	France	Metabolic (0.5)<br>Structural pools (3)<br>Active SOM (1.5)<br>Slow SOM (25)<br>Passive SOM (1000)	Grassland and livestock	x	x		x	x		x		x	hour	Plant growth, fertiliser	Temp., Precip., radiation, water vapour, depth, pH, BD, texture (clay, silt, sand), mowing, grazing dates, animal type	x		C, N, water and energy and GHG flows	https://www6.ara.inrae.fr/urep/Nos-ressources/Plateforme-modelisation/PaSim
STICS [118,119]	France	humified organic matter<br>crop residues<br>microbial biomass	Cropland	x	x	x			x	x			day	SOM, fertilisation,	Min and max Temp., Precip., sowing dates and densities, irrigation, rotations, harvesting methods (harvesting, picking, mowing, etc.)			C change, Yield, quality of the harvested organs (e.g. sugar content), crop water	https://www6.paca.inrae.fr/stics

Model	Original location	C pools (residence time in years)	Land use type	Spatial resolution						Temporal resolution				C inputs	Parameters influencing mineralisation	C:N	14C	C output (soil depth in cm)	Download/ documentation URL
				P	F	CT	RG	NA	GL	S	M	L	step						
																		use, N leaching, N_2O emissions	
ORCHIDEE water-energy-carbon budget [120]		Active (0.5-1) Slow (10-50) Passive (400-4000)	Cropland, Grassland, Forest land, Bare soils				x	x	x	x			½ hour, day	Litter, fertiliser	Temp., Precip., solar radiation, soil moisture, clay, surface air pressure, wind, humidity, atmospheric CO_2			C dynamic, GHG emissions (CO_2, H_2O), heat exchange	https://orchidas.lsce.ipsl.fr/

Abbreviations: P: Plot, F: Field, CT: Catchman, RG: Regional, NA: National, GB: Global, S: short-term, M: medium-term; L: long-term, Temp.: temperature, Precip.: precipitation, EVT: Evapotranspiration, SOC: soil organic carbon, BD bulk density.

RothC initialisation

To initialize the RothC model, we applied the pedotransfer functions for the three active RothC pools by Weihermüller et al. [121] (Eq. 1 to Eq. 3) and the function by Falloon et al. [122] for the IOM pool (Eq. 4). All these functions are based on regression analyses.

$$RPM = (0.1847 \times SOC + 0.1555)(clay + 1.2750)^{-0.1158} \quad \textbf{Eq. 1}$$

$$HUM = (0.7148 \times SOC + 0.5069)(clay + 0.3421))^{0.0184} \quad \textbf{Eq. 2}$$

$$BIO = (0.0140 \times SOC + 0.0075)(clay + 8.8473)^{0.0567} \quad \textbf{Eq. 3}$$

$$IOM = 0.049 \times SOC^{1.139} \quad \textbf{Eq. 4}$$

where SOC and clay are expressed in t ha^{-1} and % respectively. The IOM function in absence of radiocarbon data based on clay content and/or SOC. The DPM:RPM ratio for C inputs from plant residues per crop, grass, and tree cover was set at 1.44 (59% RPM and 41% DPM), 0.67 and 0.25 respectively [85]. The BIO:HUM ratio was set at 0.0259, 0.0272 and 0.0261 for temperate grass, crop and alpine cover respectively [91]. The decay constant k [yr^{-1}] for DPM, RPM, BIO and HUM was set at 10, 0.3, 0.66 and 0.02 respectively [121].

SOC erosion

Eroded soil organic carbon (SOC_{eroded}) [Mg SOC ha^{-1} yr^{-1}] is calculated following the method in [123] (Eq. 5).

$$SOC_{eroded} = SOC \times \left[\frac{soil\ erosion}{(bulk\ desity\ \times depth)}\right] \times ER\ \times CF \quad \textbf{Eq. 5}$$

where SOC, bulk density and depth are available from the Harmonized World Soil Database (HWSD) [6], ER (enrichment factor) is set to 1 and CF (cover-management factor) is a species-dependent factor listed in Table S9.

Table S9. Mean cover-management factors (C-factors) [dimensionless] per non-arable land-cover types and crop types at European and global scales

Group	EU	World
Forest	0.0001-0.003	0.0001-0.003
Permanent crops	0.1-0.3	
Pastures/Grassland	0.05-0.15	0.01-0.15
Scrub/Shrubland	0.01-0.1	0.01-0.15
Arable	0.233	
Arable no conservation management	1.23	
Arable conservation management	0.809	
Arable conservation tillage	0.83	
Use of crop residues	0.9888	
use of cover crops	0.987	
Savanna		0.01-0.15
Trees/fruit trees		0.15
Cereals		0.1
Fibre crops		0.28
Roots/tuber		0.34

Source: EU [124], World [11]

For reference, annual averaged soil loss by erosion (E) [t ha^{-1} yr^{-1}] was computed in the input data source (GloSEM v1.1) with the RUSLE2015 equation [125], as modified from the original RUSLE [126] (Eq. 6).

$$E = R \times K \times C \times LS \times P \qquad \textbf{Eq. 6}$$

where R the rainfall erosivity factor [MJ mm ha^{-1} ha^{-1} yr^{-1}], and K is soil erodibility factor [t ha h ha^{-1} MJ^{-1} mm^{-1}], C is the cover-management factor (dimensionless), LS is slope length and slope steepness factor (dimensionless), and P is support practices factor (dimensionless).

References

1. GADM. Global Administrative (GADM)maps and data. https://gadm.org/download_world.html (2018).
2. FAO and ITPS. *Global Soil Organic Map V1.5: Technical Report*. (2020) doi:https://doi.org/10.4060/ca7597en.
3. ESA. Land Cover CCI Product User Guide Version 2. Tech. Rep. maps.elie.ucl.ac.be/CCI/viewer/download/ESACCI-LC-Ph2-PUGv2_2.0.pdf (2017).
4. Di Gregorio, A. *Land Cover Classification System. Classification concepts. Software version 3*. *October* (2016).
5. UNEP-WCMC. *User Manual for the World Database on Protected Areas and world database on other effective area- based conservation measures : 1.6*. https://wdpa.s3-eu-west-1.amazonaws.com/WDPA_Manual/English/WDPA_WDOECM_Manual_1_6.pdf (2019).
6. FAO/IIASA. *Harmonized World Soil Database (version 1.2)*. *FAO, Rome, Italy and IIASA, Laxenburg, Austria* (2009).
7. Danielson, J. J. . & Gesch, D. B. *Global multi-resolution terrain elevation data 2010 (GMTED2010)*. vol. 2010 (2011).
8. Karger, D. N. *et al.* Climatologies at high resolution for the earth's land surface areas. *Sci. Data* **4**, 1–20 (2017).
9. Karger, D. N. *et al.* Data from: Climatologies at high resolution for the earth's land surface areas. *Dryad Digit. Repos.* (2018) doi:http://dx.doi.org/doi:10.5061/dryad.kd1d4.
10. FAO. *Global ecological zones for FAO forest reporting: 2010 Update*. *Forest resources Assessment Working Paper 179* (2012).
11. Borrelli, P. *et al.* An assessment of the global impact of 21st century land use change on soil erosion. *Nat. Commun.* **8**, (2013).
12. Trabucco, A. & Zomer, R. J. *Global High-Resolution Soil-Water Balance. figshare. Dataset*. vol. 2010 (2010).
13. FAO. FAO ECOCROP: The Crop Environmental Requirements Database. http://ecocrop.fao.org/ecocrop/srv/en/home; available on: https://github.com/supersistence/EcoCrop-ScrapeR (2018).
14. FAO. FAOSTAT Crops. http://www.fao.org/faostat/en/#data/QC (2020).
15. Li, W., Ciais, P., Makowski, D. & Peng, S. Data descriptor: A global yield dataset for major lignocellulosic bioenergy crops based on field measurements. *Sci. Data* **5**, 1–10 (2018).
16. Cossel, M. Von *et al.* Marginal Agricultural Land Low-Input Systems for Biomass Production. *Energies* **12**, 0–25 (2019).
17. Elbersen, B. *et al. Deliverable 2.6 Methodological approaches to identify and map marginal land suitable for industrial crops in Europe*. *EU Horizon 2020; MAGIC; GA-No.: 727698* (2020).
18. Mellor, P., Lord, R. A., Joao, E., Thomas, R. & Hursthouse, A. Identifying non-agricultural marginal lands as a route to sustainable bioenergy provision - A review and holistic definition. *Renew. Sustain. Energy Rev.* **135**, (2020).
19. Rettenmaier, N., Schorb, A., Hienz, G. & Diaz-Chavez, R. A. *Report on sustainability impacts of the*

use of marginal areas and grassy biomass (D 5.4). (2012).

20. Smit, B., Bray, J. & Keddie, P. Identification of marginal agricultural areas in Ontario, Canada. *Geoforum* **22**, 333–346 (1991).

21. CGIAR TAC. *CGIAR Research Priorities for Marginal Lands*. https://cgspace.cgiar.org/handle/10947/332 (2000).

22. Kang, S. *et al.* Marginal Lands: Concept, Assessment and Management. *J. Agric. Sci.* **5**, 129–139 (2013).

23. Gelfand, I. *et al.* Sustainable bioenergy production from marginal lands in the US Midwest. *Nature* **493**, 514–517 (2013).

24. Milbrandt, A. & Overend, R. P. Assessment of Biomass Resources from Marginal Lands in APEC Economies. 52 (2009) doi:10.2172/968464.

25. Blanco-Canqui, H. Growing Dedicated Energy Crops on Marginal Lands and Ecosystem Services. *Soil Sci. Soc. Am. J.* **80**, 845–858 (2016).

26. Dale, B. E., Bals, B. D., Kim, S. & Eranki, P. Biofuels done right: Land efficient animal feeds enable large environmental and energy benefits. *Environ. Sci. Technol.* **44**, 8385–8389 (2010).

27. Gibbs, H. K. & Salmon, J. M. Mapping the world's degraded lands. *Appl. Geogr.* **57**, 12–21 (2015).

28. Olsson, L. *et al.* Land Degradation. in *Climate Change and Land: an IPCC special report on climate change, desertification, land degradation, sustainable land management, food security, and greenhouse gas fluxes in terrestrial ecosystems* (ed. P.R. Shukla, J. Skea, E. Calvo Buendia, V. Masson-Delmotte, H.-O. Pörtner, D. C. Roberts, P. Zhai, R. Slade, S. Connors, R. van Diemen, M. Ferrat, E. Haughey, S. Luz, S. Neogi, M. Pathak, J. Petzold, J. Portugal Pereira, P. Vyas, E. Huntley, K. Kissick, M, J. M.) 345–436 (2019). doi:10.1002/9781118786352.wbieg0538.

29. Jones, R. *et al. Updated common bio-physical criteria to define natural constraints for agriculture in Europe : definition and scientific justification for the common biophysical criteria : technical factsheets.* (2012). doi:10.2788/91182.

30. Eliasson, Å. *Review of Land Evaluation Methods for Quantifying Natural Constraints to Agriculture*. *JRC Scientific and Technical Reports* (2007).

31. Galland, V., Avadí, A. & Bockstaller, C. Data to inform the modelling of direct nitrogen field emissions from global agriculture. *Data Br.* (2020).

32. Gopalakrishnan, G. *et al.* Biofuels, land, and water: A systems approach to sustainability. *Environ. Sci. Technol.* **43**, 6094–6100 (2009).

33. Fiorese, G. & Guariso, G. A GIS-based approach to evaluate biomass potential from energy crops at regional scale. *Environ. Model. Softw.* **25**, 702–711 (2010).

34. Hollander, J. H. The Concept of Marginal Rent. *Q. J. Econ.* **9**, 175–187 (1895).

35. Strijker, D. Marginal lands in Europe - Causes of decline. *Basic Appl. Ecol.* **6**, 99–106 (2005).

36. WBGU. *World in Transition – Future Bioenergy and Sustainable Land Use*. *Management of Environmental Quality: An International Journal* vol. 21 (2008).

37. Campbell, J. E., Lobell, D. B., Genova, R. C. & Field, C. B. The global potential of bioenergy on abandoned agriculture lands. *Environ. Sci. Technol.* **42**, 5791–5794 (2008).

38. Kukk, L. *et al.* Assessment of abandoned agricultural land resource for bio-energy production in Estonia. *Acta Agric. Scand. Sect. B — Soil Plant Sci.* **60**, 166–173 (2010).

39. Cai, X., Zhang, X. & Wang, D. Land Availability Analysis for Biofuel Production. *Environ. Sci. Technol.* **45**, 334–339 (2011).

40. Wicke, B. Bioenergy Production on Degraded and Marginal Land: Assessing its potentials, economic

performance, and environmental impacts for different settings and geographical scales. **PhD**, 203 (2011).

41. Odeh, I. O. A., Tan, D. K. Y. & Ancev, T. Potential Suitability and Viability of Selected Biodiesel Crops in Australian Marginal Agricultural Lands Under Current and Future Climates. *BioEnergy Res.* **4**, 165–179 (2011).

42. Swinton, S. M., Babcock, B. A., James, L. K. & Bandaru, V. Higher US crop prices trigger little area expansion so marginal land for biofuel crops is limited. *Energy Policy* **39**, 5254–5258 (2011).

43. Dauber, J. *et al.* Bioenergy from 'surplus' land: Environmental and socio-economic implications. *BioRisk* **50**, 5–50 (2012).

44. Fahd, S., Fiorentino, G., Mellino, S. & Ulgiati, S. Cropping bioenergy and biomaterials in marginal land: The added value of the biorefinery concept. *Energy* **37**, 79–93 (2012).

45. Liu, L., Zhuang, D., Jiang, D. & Huang, Y. Assessing the potential of the cultivation area and greenhouse gas (GHG) emission reduction of cassava-based fuel ethanol on marginal land in Southwest China. *African J. Agric. Res.* **7**, 5594–5603 (2012).

46. Lu, L., Jiang, D., Zhuang, D. & Huang, Y. Evaluating the marginal land resources suitable for developing Pistacia chinensis-based biodiesel in China. *Energies* **5**, 2165–2177 (2012).

47. Shortall, O. K. 'Marginal land' for energy crops: Exploring definitions and embedded assumptions. *Energy Policy* **62**, 19–27 (2013).

48. Niblick, B., Monnell, J. D., Zhao, X. & Landis, A. E. Using geographic information systems to assess potential biofuel crop production on urban marginal lands. *Appl. Energy* **103**, 234–242 (2013).

49. Milbrandt, A. R., Heimiller, D. M., Perry, A. D. & Field, C. B. Renewable energy potential on marginal lands in the United States. *Renew. Sustain. Energy Rev.* **29**, 473–481 (2014).

50. Saha, M. & Eckelman, M. J. Geospatial assessment of potential bioenergy crop production on urban marginal land. *Appl. Energy* **159**, 540–547 (2015).

51. Xue, S., Lewandowski, I., Wang, X. & Yi, Z. Assessment of the production potentials of Miscanthus on marginal land in China. *Renew. Sustain. Energy Rev.* **54**, 932–943 (2016).

52. Dauber, J. & Miyake, S. To integrate or to segregate food crop and energy crop cultivation at the landscape scale? Perspectives on biodiversity conservation in agriculture in Europe. *Energy. Sustain. Soc.* **6**, (2016).

53. Gerwin, W. *et al.* Assessment and quantification of marginal lands for biomass production in Europe using soil-quality indicators. *Soil* **4**, 267–290 (2018).

54. Saha, M. & Eckelman, M. J. Geospatial assessment of regional scale bioenergy production potential on marginal and degraded land. *Resour. Conserv. Recycl.* **128**, 90–97 (2018).

55. Schröder, P. *et al.* Intensify production, transform biomass to energy and novel goods and protect soils in Europe—A vision how to mobilize marginal lands. *Sci. Total Environ.* **616**–**617**, 1101–1123 (2018).

56. IIASA/FAO. *Global Agro-ecological Zones (GAEZ v3.0)*. (2012).

57. FAO. *Global Soil Organic Carbon Map (GSOCmap) Technical Report*. (2018).

58. Ledo, A. *et al.* A global, empirical, harmonised dataset of soil organic carbon changes under perennial crops. *Sci. Data* **6**, 1–7 (2019).

59. Köppen, W. *Grundrisse der Klimakunde*. (Walter de Gruyter Co., 1931).

60. Trewartha, G. T. *An introduction to climate*. (Mc Graw-Hill, 1968).

61. Bolinder, M. A., Janzen, H. H., Gregorich, E. G., Angers, D. A. & VandenBygaart, A. J. An approach for estimating net primary productivity and annual carbon inputs to soil for common agricultural crops

in Canada. *Agric. Ecosyst. Environ.* **118**, 29–42 (2007).

62. Monfreda, C., Ramankutty, N. & Foley, J. A. Farming the planet: 2. Geographic distribution of crop areas, yields, physiological types, and net primary production in the year 2000. *Global Biogeochem. Cycles* **22**, 1–19 (2008).

63. Ronzon, T., Piotrowski, S. & Carus, M. DataM – Biomass estimates (v3): a new database to quantify biomass availability in the European Union. *JRC Tech. Rep.* (2015) doi:10.2791/650215.

64. Ma, S. *et al.* Variations and determinants of carbon content in plants: A global synthesis. *Biogeosciences* **15**, 693–702 (2018).

65. Smith, P. *et al.* How to measure, report and verify soil carbon change to realize the potential of soil carbon sequestration for atmospheric greenhouse gas removal. *Glob. Chang. Biol.* **26**, 219–241 (2020).

66. Pausch, J. & Kuzyakov, Y. Carbon input by roots into the soil: Quantification of rhizodeposition from root to ecosystem scale. *Glob. Chang. Biol.* **24**, 1–12 (2018).

67. Alexopoulou, E. *D1.3: List with the selected most promising industrial crops for marginal lands*. https://magic-h2020.eu/ (2018).

68. Mathew, I., Shimelis, H., Mutema, M. & Chaplot, V. What crop type for atmospheric carbon sequestration: Results from a global data analysis. *Agric. Ecosyst. Environ.* **243**, 34–46 (2017).

69. Von Cossel, M. *et al. Deliverable 4.1: Low-input agricultural practices for industrial crops on marginal land*. (2020).

70. FAO. *Measuring and modelling soil carbon stocks and stock changes in livestock production systems: Guidelines for assessment (Version 1). Livestock Environmental Assessment and Performance (LEAP) Partnership*. (2019).

71. Campbell, E. E. & Paustian, K. Current developments in soil organic matter modeling and the expansion of model applications: a review. *Environ. Res. Lett.* **10**, 123004 (2015).

72. Manzoni, S. & Porporato, A. Soil carbon and nitrogen mineralization: Theory and models across scales. *Soil Biol. Biochem.* **41**, 1355–1379 (2009).

73. Hénin, S. & Dupuis, M. Essai de bilan de la matière organique du sol. *Ann. Agron.* **1**, 19–29 (1945).

74. Smith, P. *et al.* How to measure, report and verify soil carbon change to realize the potential of soil carbon sequestration for atmospheric greenhouse gas removal. *Glob. Chang. Biol.* **26**, 219–241 (2020).

75. Paustian, K., Larson, E., Kent, J., Marx, E. & Swan, A. Soil C Sequestration as a Biological Negative Emission Strategy. *Front. Clim.* **1**, 1–11 (2019).

76. Köck, K., Leifeld, J. & Fuhrer, J. *A model-based inventory of sinks and sources of CO2 in agricultural soils in Switzerland : development of a concept*. (2013).

77. IPCC. *IPCC guidelines for national greenhouse gas inventories. Chapter 4. agriculture, forestry and other land use*. *IPCC* http://www.ipcc-nggip.iges.or.jp/public/2006gl/pdf/4_Volume4/V4_04_Ch4_Forest_Land.pdf (2006).

78. FAO. *Technical specifications and country guidelines for Global Soil Organic Carbon Sequestration Potential Map GSOCseq*. *NASPA Journal* vol. 42 (2020).

79. Petersen, B. M., Olesen, J. E. & Heidmann, T. A flexible tool for simulation of soil carbon turnover. *Ecol. Modell.* **151**, 1–14 (2002).

80. Falloon, P. & Smith, P. Simulating SOC changes in long-term experiments with rothC and CENTURY: Model evaluation for a regional scale application. *Soil Use Manag.* **18**, 101–111 (2002).

81. Cerri, C. E. P. *et al.* Predicted soil organic carbon stocks and changes in the Brazilian Amazon between 2000 and 2030. *Agric. Ecosyst. Environ.* **122**, 58–72 (2007).

82. Coleman, K. *et al.* Simulating trends in soil organic carbon in long-term experiments using RothC-26.3. *Geoderma* **81**, 29–44 (1997).

83. Jenkinson, D. S. & Celeman, K. Calculating the annual input of organic matter to soil from measurements of total organic carbon and radiocarbon. *Eur. J. Soil Sci.* **45**, 167–174 (1994).

84. Coleman, K. & Jenkinson, D. S. *RothC - A model for the turnover of carbon in soil. Model description and users guide (updated June 2014)*. *Rothamsted Research* https://www.rothamsted.ac.uk/sites/default/files/RothC_guide_WIN.pdf (2014).

85. Gottschalk, P. *et al.* How will organic carbon stocks in mineral soils evolve under future climate? Global projections using RothC for a range of climate change scenarios. *Biogeosciences* **9**, 3151–3171 (2012).

86. Morais, T. G., Teixeira, R. F. M. & Domingos, T. Detailed global modelling of soil organic carbon in cropland, grassland and forest soils. *PLoS One* **14**, 1–27 (2019).

87. Falloon, P. *et al.* RothCUK - A dynamic modelling system for estimating changes in soil C from mineral soils at 1-km resolution in the UK. *Soil Use Manag.* **22**, 274–288 (2006).

88. Jenkinson, D. S. & Coleman, K. The turnover of organic carbon in subsoils. Part 2. Modelling carbon turnover. *Eur. J. Soil Sci.* **59**, 400–413 (2008).

89. Shirato, Y., Hakamata, T. & Taniyama, I. Modified rothamsted carbon model for andosols and its validation: changing humus decomposition rate constant with pyrophosphate-extractable Al. *Soil Sci. Plant Nutr.* **50**, 149–158 (2004).

90. Farina, R., Coleman, K. & Whitmore, A. P. Modification of the RothC model for simulations of soil organic C dynamics in dryland regions. *Geoderma* **200**–**201**, 18–30 (2013).

91. Zimmermann, M., Leifeld, J., Schmidt, M. W. I., Smith, P. & Fuhrer, J. Measured soil organic matter fractions can be related to pools in the RothC model. *Eur. J. Soil Sci.* **58**, 658–667 (2007).

92. IPCC. Chapter 4. Agriculture, Forestry and other Land Use. in *2006 IPCC Guidelines for National Greenhouse Gas Inventories* (eds. Eggleston, S., Buendia, L., Miwa, K., Ngara, T. & Tanabe, K.) (Intergovernmental Panel on Climate Change, Prepared by the National Greenhouse Gas Inventories Programme, 2006).

93. Andriulo, A. *et al.* Modelling soil carbon dynamics with various cropping sequences on the rolling pampas. *Agron. EDP Sci.* **19**, 365–377 (1999).

94. Saffih-Hdadi, K. & Mary, B. Modeling consequences of straw residues export on soil organic carbon. *Soil Biol. Biochem.* **40**, 594–607 (2008).

95. Clivot, H. *et al.* Modeling soil organic carbon evolution in long-term arable experiments with AMG model. *Environ. Model. Softw.* **118**, 99–113 (2019).

96. Andrén, O. & Kätterer, T. ICBM: The introductory carbon balance model for exploration of soil carbon balances. *Ecol. Appl.* **7**, 1226–1236 (1997).

97. Andrén, O., Kätterer, T. & Karlsson, T. ICBM regional model for estimations of dynamics of agricultural soil carbon pools. *Nutr. Cycl. Agroecosystems* **70**, 231–239 (2004).

98. Petersen, B. M. *C-TOOL version 1.1. A tool for simulation of soil carbon turnover. Description and users guide*. (2003).

99. Taghizadeh-Toosi, A. *et al.* C-TOOL: A simple model for simulating whole-profile carbon storage in temperate agricultural soils. *Ecol. Modell.* **292**, 11–25 (2014).

100. Petersen, B. M., Berntsen, J., Hansen, S. & Jensen, L. S. CN-SIM — a model for the turnover of soil organic matter. I. Long-term carbon and radiocarbon development. *Soil Biol. Biochem.* **37**, 359–374 (2005).

101. Molina, J. A. E. Description of the model NCSOIL. in *Evaluation of Soil Organic Matter Models* (eds.

Powlson., D. S., Smith, P. & Smith, J. U.) vol. 1 269–274 (Springer-Verlag, 1996).

102. Liski, J., Palosuo, T., Peltoniemi, M. & Sievänen, R. Carbon and decomposition model Yasso for forest soils. *Ecol. Modell.* **189**, 168–182 (2005).

103. Järvenpää, M., Repo, A., Akujärvi, A., Kaasalainen, M. & Liski, J. Soil carbon model Yasso15 - Bayesian calibration using worldwide litter decomposition and carbon stock data (MANUSCRIPT IN PREPARATION). 1–19 (2018).

104. Chertov, O. & Komarov, A. SOMM: A model of soil organic matter dynamics. *Ecol. Modell.* **94**, 177–189 (1997).

105. Grace, P. R., Ladd, J. N., Robertson, G. P. & Gage, S. H. SOCRATES-A simple model for predicting long-term changes in soil organic carbon in terrestrial ecosystems. *Soil Biol. Biochem.* **38**, 1172–1176 (2006).

106. Parton, W. J., Stewart, J. W. B. & Cole, C. V. Dynamics of C , N , P and S in grassland soils: a model. *Biogeochemistry* **131**, 109–131 (1988).

107. Metherell, A. K., Harding, L. A., Cole, C. V. & Parton, W. J. *CENTURY Soil Organic Matter Model Environment: Technical Documentation Agroecosystem Version 4.0*. (1993).

108. Smith, J. *et al. Model to Estimate Carbon in Organic Soils – Sequestration and Emissions (ECOSSE). User- Manual*. vol. 44 http://www.abdn.ac.uk/ibes/staff/jo.smith/ECOSSE (2010).

109. Parton, W. J., Ojima, D. S., Cole, C. V. & Schimel, D. S. A General Model for Soil Organic Matter Dynamics: Sensitivity to Litter Chemistry, Texture and Management. *Quantitative Modeling of Soil Forming Processes* 147–167 (1994) doi:https://doi.org/10.2136/sssaspecpub39.c9.

110. Del Grosso, S. J. *et al.* Simulated interaction of carbon dynamics and nitrogen trace gas fluxes using the DAYCENT model. in *Modeling Carbon and Nitrogen Dynamics for Soil Management.* (eds. M. Schaffer, M., L., Ma, L. S. & Hansen, S.) 303–332 (2001).

111. Del Grosso, S. J. *et al.* Global scale DAYCENT model analysis of greenhouse gas emissions and mitigation strategies for cropped soils. *Glob. Planet. Change* **67**, 44–50 (2009).

112. Li, C., Frolking, S. & Frolking, T. A Model of Nitrous Oxide Evolution From Soil Driven by Rainfall Events. 1. Model Structure and Sensitivity. *J. Geophys. Res.* **97**, 9759–9776 (1992).

113. Sharpley, A. N. & Williams, J. R. EPIC: The erosion-productivity impact calculator. *U.S. Dep. Agric. Tech. Bull.* 235 (1990).

114. Hansen, S., Abrahamsen, P., T. Petersen, C. & Styczen, M. Daisy: Model Use, Calibration, and Validation. *Trans. ASABE* **55**, 1317 (2012).

115. Franko, U. *et al.* Simulating trends in soil organic carbon in long-term experiments using the CANDY model. *Geoderma* **81**, 5–28 (2002).

116. Kuka, K., Franko, U. & Rühlmann, J. Modelling the impact of pore space distribution on carbon turnover. *Ecol. Modell.* **8**, 295–306 (2007).

117. Brilli, L. *et al.* Review and analysis of strengths and weaknesses of agro-ecosystem models for simulating C and N fluxes. *Sci. Total Environ.* **598**, 445–470 (2017).

118. Brisson, N. *et al.* An overview of the crop model STICS. *Eur. J. Agron.* **18**, 309–332 (2003).

119. Brisson, N. *et al.* STICS : a generic model for the simulation of crops and their water and nitrogen balances . I . Theory and parameterization applied to wheat and corn. *Agronomic* **18**, 311–346 (1998).

120. Krinner, G. *et al.* A dynamic global vegetation model for studies of the coupled atmosphere-biosphere system. *Global Biogeochem. Cycles* **19**, (2005).

121. Weihermüller, L., Graf, A., Herbst, M. & Vereecken, H. Simple pedotransfer functions to initialize reactive carbon pools of the RothC model. *Eur. J. Soil Sci.* **64**, 567–575 (2013).

122. Falloon, P., Smith, P., Coleman, K. & Marshall, S. Estimating the size of the inert organic matter pool from total soil organic carbon content for use in the Rothamsted carbon model. *Soil Biol. Biochem.* **30**, 1207–1211 (1998).

123. Lugato, E., Paustian, K., Panagos, P., Jones, A. & Borrelli, P. Quantifying the erosion effect on current carbon budget of European agricultural soils at high spatial resolution. *Glob. Chang. Biol.* **22**, 1976–1984 (2016).

124. Panagos, P. *et al.* Estimating the soil erosion cover-management factor at the European scale. *Land use policy* **48**, 38–50 (2015).

125. Panagos, P. *et al.* The new assessment of soil loss by water erosion in Europe. *Environ. Sci. Policy* **54**, 438–447 (2015).

126. Foster, R. G. *Revised Universal Soil Loss Equation – Version 2 (RUSLE2)*. (2005).
