# Supplementary material for: A generalizable framework for spatially explicit exploration of soil organic carbon sequestration on global marginal land
Source: Sci Rep. 2022 Jul 1;12:11144. doi: 10.1038/s41598-022-14759-w (PMC9249906; doi:10.1038/s41598-022-14759-w)
Supplement: Supplementary file 2 — Supplementary Results. [file 41598_2022_14759_MOESM2_ESM.pdf]

Supplementary Results for:
A generalizable framework for spatially explicit exploration of soil organic carbon sequestration on global marginal land

Ariane Albers[1,*], Angel Avadí[2,3], Lorie Hamelin[1]

[1] TBI, Université de Toulouse, CNRS, INRAE, INSA, Toulouse, France
[2] CIRAD, UPR Recyclage et risque, F-34398 Montpellier, France
[3] Univ Montpellier, CIRAD, Montpellier, France
*Corresponding author: albers.ariane@gmail.com

Contents

Maps of global target areas 2
Identified target areas (marginal land) 9
Potential SOC changes associated with plant species and land use change 11
References 15

List of figures

Figure S1. Global SOC map by SOC content class. 2
Figure S2. Global map of sparsely vegetated and bare areas. 2
Figure S3. Selected local maps of abandoned agricultural land - Europe. 3
Figure S4. Selected local maps of abandoned agricultural land – North America and Asia. 4
Figure S5. Selected local maps of abandoned agricultural land – Africa. 5
Figure S6. Selected local maps of abandoned agricultural land – South America. 6
Figure S7. Global map of officially protected land and coastal areas. 7
Figure S8. Global map of biophysical soil constraints to plants. 7
Figure S9. Global map of FAO's Global Ecological Zones. 8
Figure S10. Global map of World Regions 8
Figure S11. Mean annual soil organic carbon stock changes under perennial woody and herbaceous crops per climate type. 14

List of tables

Table S1 Identified marginal land up to 50 t SOC ha-1 (30 cm) per global ecological zone and world regions and corresponding top concentrations 9
Table S2. Top target area-biopump matches, area availability, and changes in soil organic carbon (SOC) between the initial (year 2020) and final (year 2100) stock levels, including SOC losses from erosion and net SOC; sorted by net SOC 10
Table S3. Mean annual soil organic carbon stock changes (ΔSOC) and standard deviation (SD) per perennial crop, climate type (tropical, subtropical, and temperate), land use change (annual crop, fallow, grassland, and forest land to perennial crop), topsoil (0-30 cm), subsoil (30-100). 11

Maps of global target areas

Figure S1 presents SOC stocks [t SOC ha^{-1}] at the topsoil layer (≤30 cm) of global areas featuring up to 50 t SOC ha^{-1}, divided into five SOC classes following increments of 10 t SOC ha^{-1}, from FAO's Global Soil Organic (GSOC v1.5) map [1] for the year 2017.

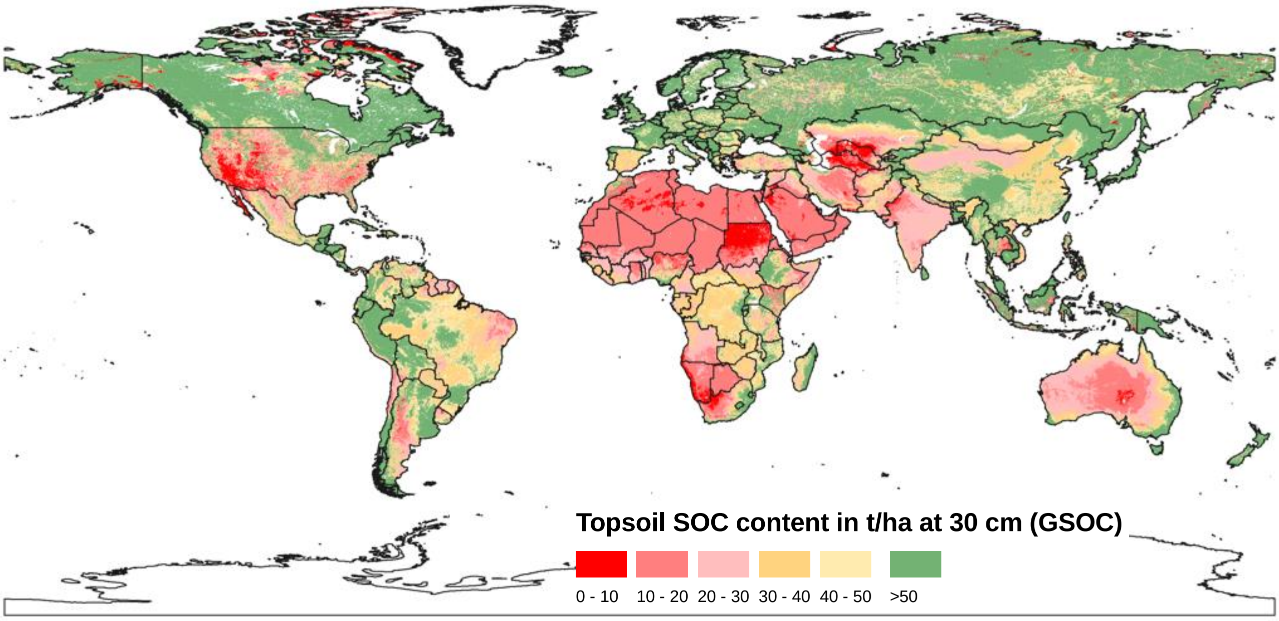

Figure S1. Global SOC map by SOC content class.

Figure S2 presents sparsely vegetated and bare areas according with FAO's Land Cover Classification System v.3 (LCCS3) [2], for the year 2018.

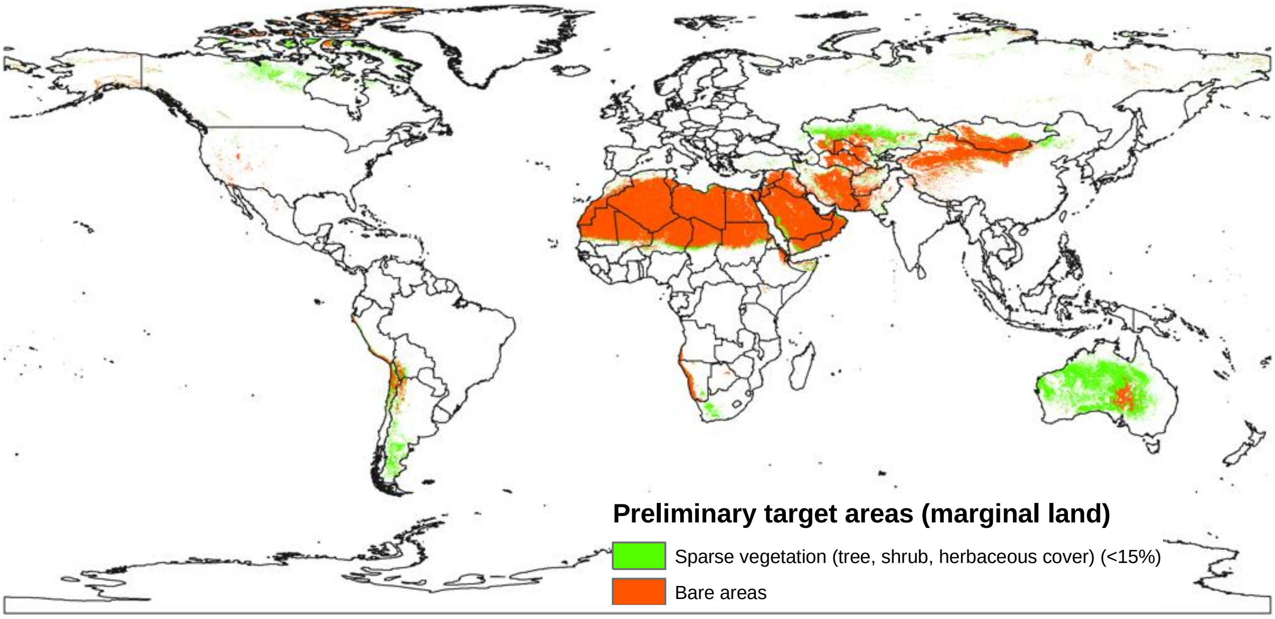

Figure S2. Global map of sparsely vegetated and bare areas.

Figure S3 to Figure S6 present local examples of agricultural land abandonment, using soil cover data from FAO's Land Cover Classification System v.3 (LCCS3) [2], representing land use changes considered as abandonment (as described in the main article) between years 2010 and 2018.

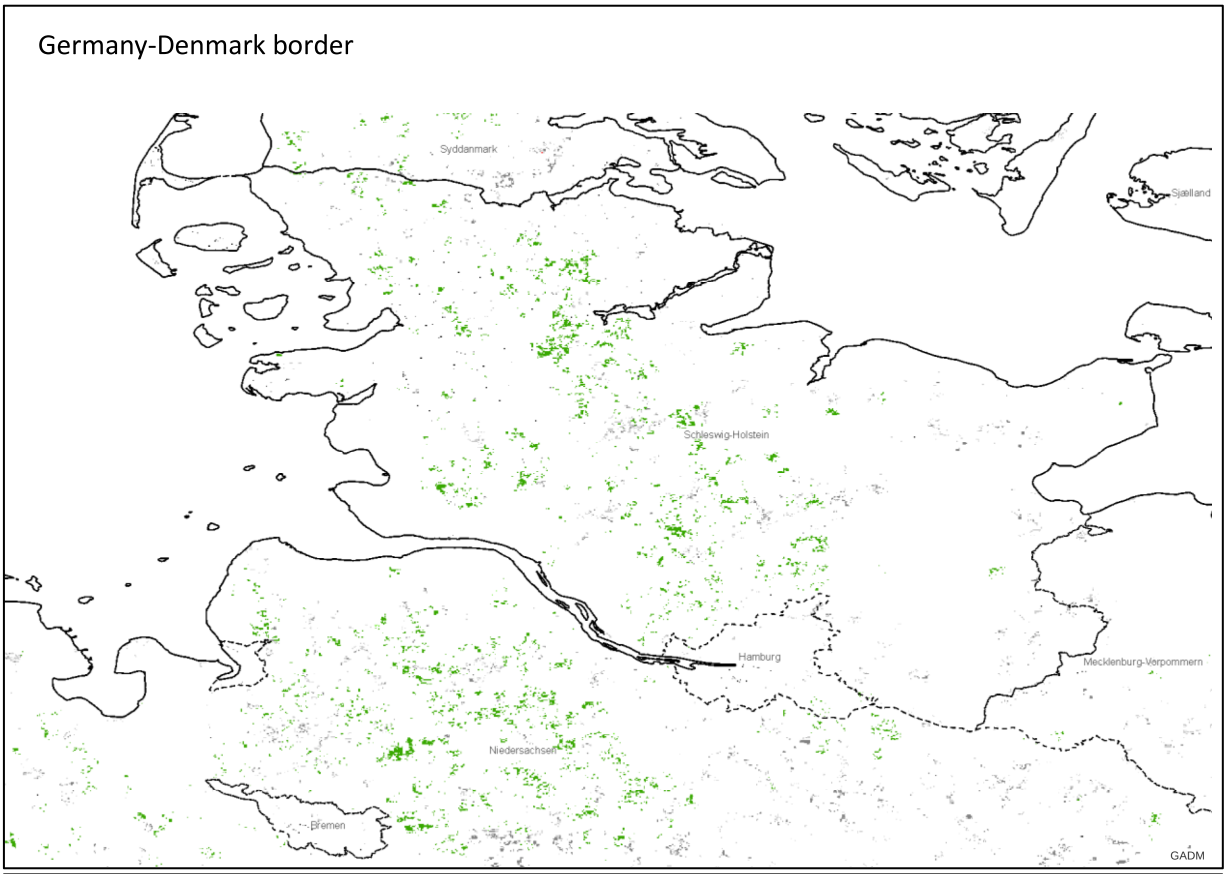

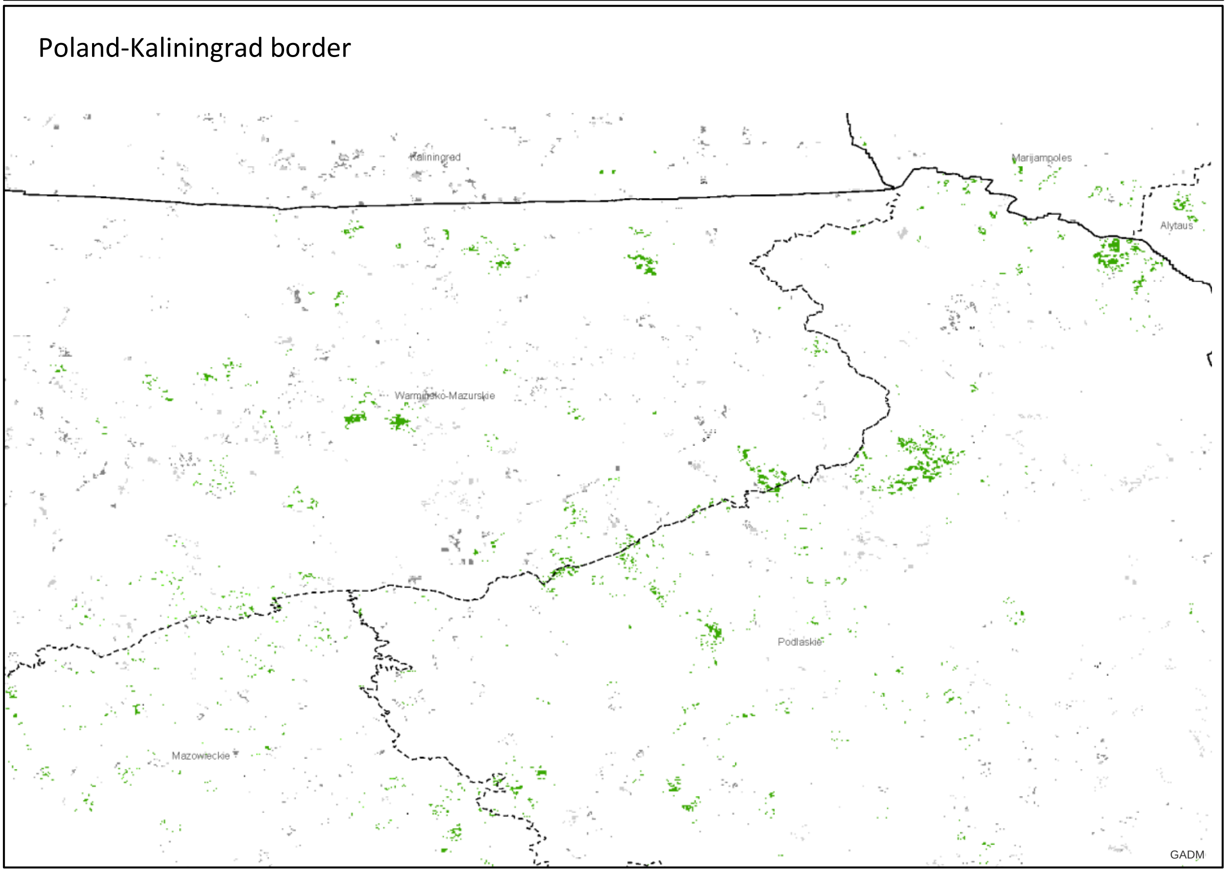

Abandoned agricultural land in 2010-2018 (LCCS3)

- No LUC
- Non-cropland to other land use
- Cropland to other land use (not abandonment)
- Non-cropland to urban
- Cropland to mosaic
- Mosaic to semi-natural
- Cropland to grassland
- Cropland to sparse vegetation
- Cropland to bare areas
- Cropland to mosaic herbaceous
- Cropland to shrubland

Figure S3. Selected local maps of abandoned agricultural land - Europe.

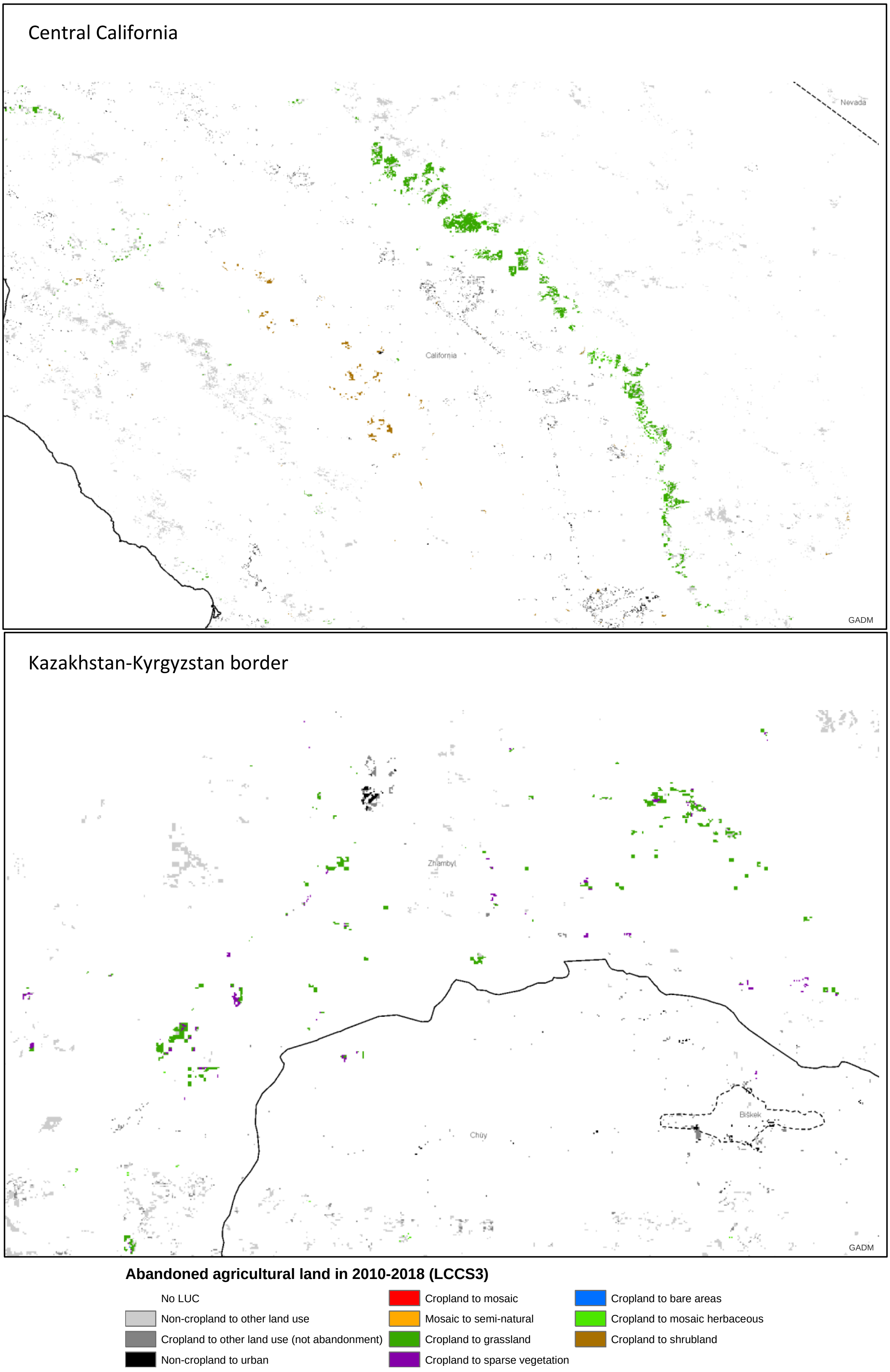

Figure S4. Selected local maps of abandoned agricultural land – North America and Asia.

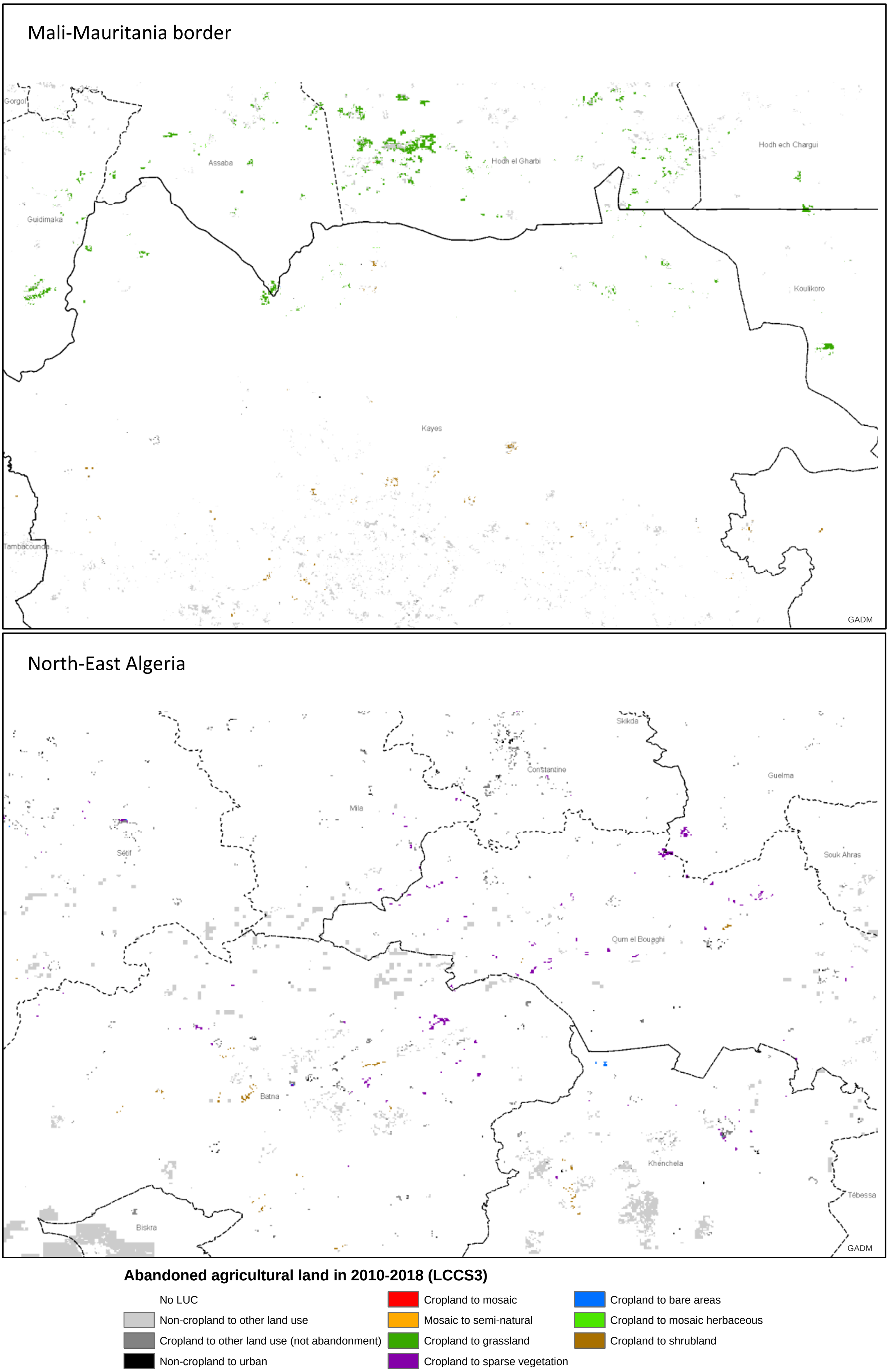

Figure S5. Selected local maps of abandoned agricultural land – Africa.

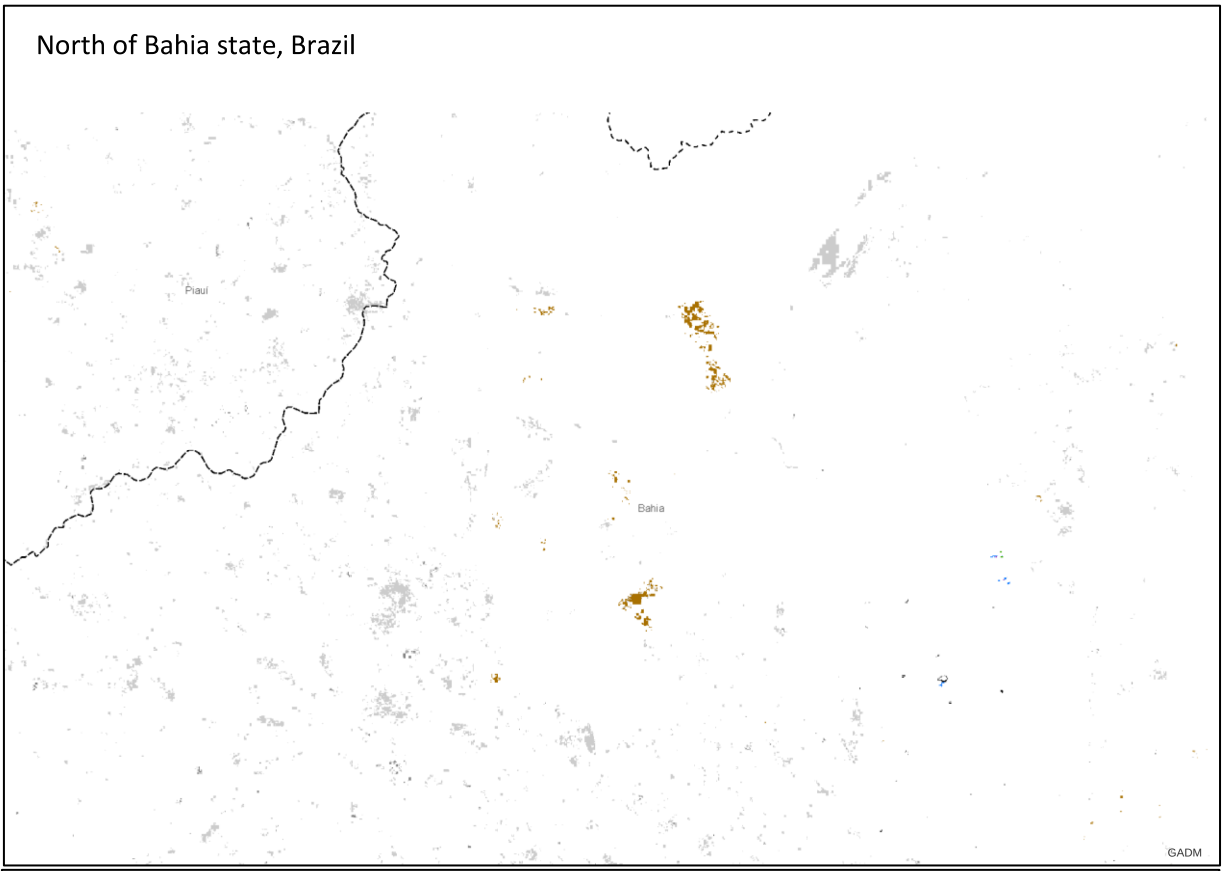

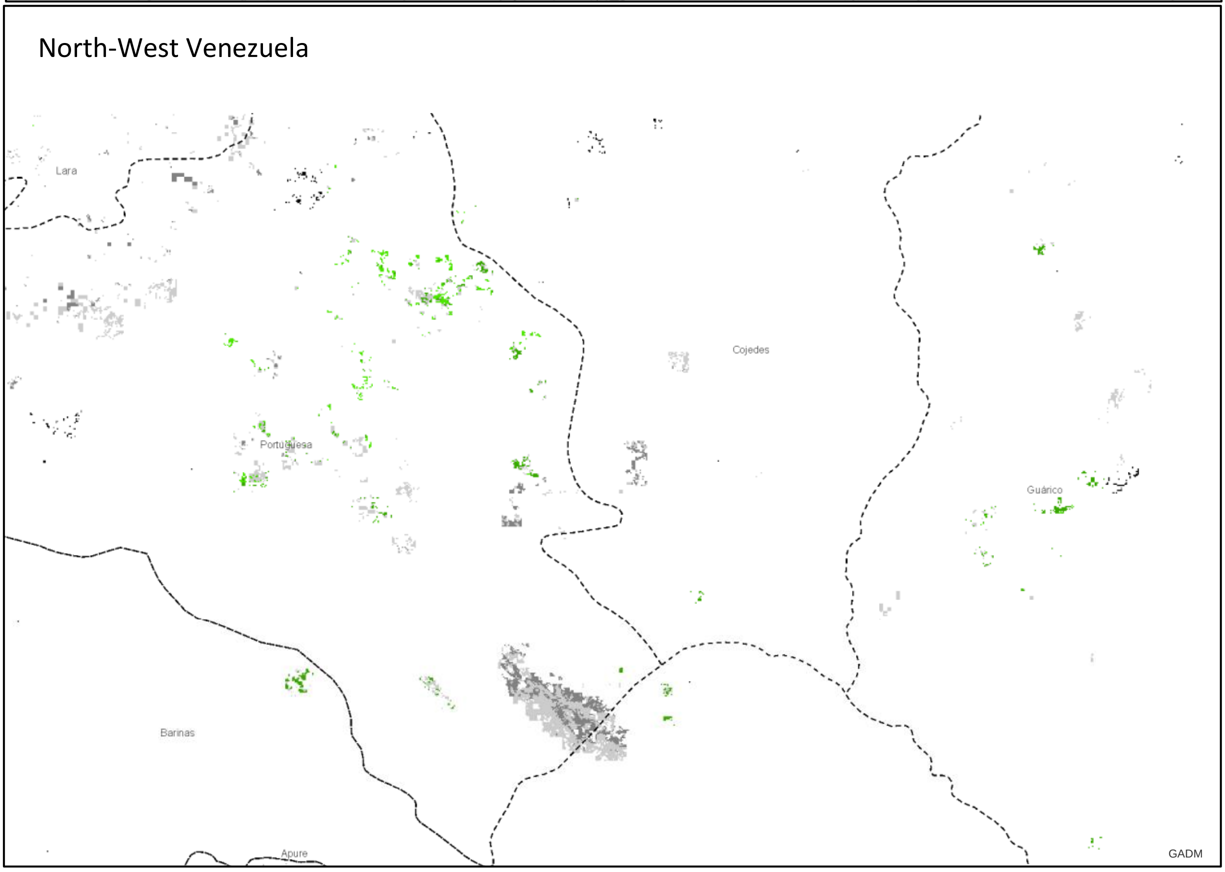

Abandoned agricultural land in 2010-2018 (LCCS3)

No LUC
Non-cropland to other land use
Cropland to other land use (not abandonment)
Non-cropland to urban
Cropland to mosaic
Mosaic to semi-natural
Cropland to grassland
Cropland to sparse vegetation
Cropland to bare areas
Cropland to mosaic herbaceous
Cropland to shrubland

Figure S6. Selected local maps of abandoned agricultural land – South America.

Figure S7 presents global protected areas according with the UN Environment Programme World Conservation Monitoring Centre [3], for the year 2016.

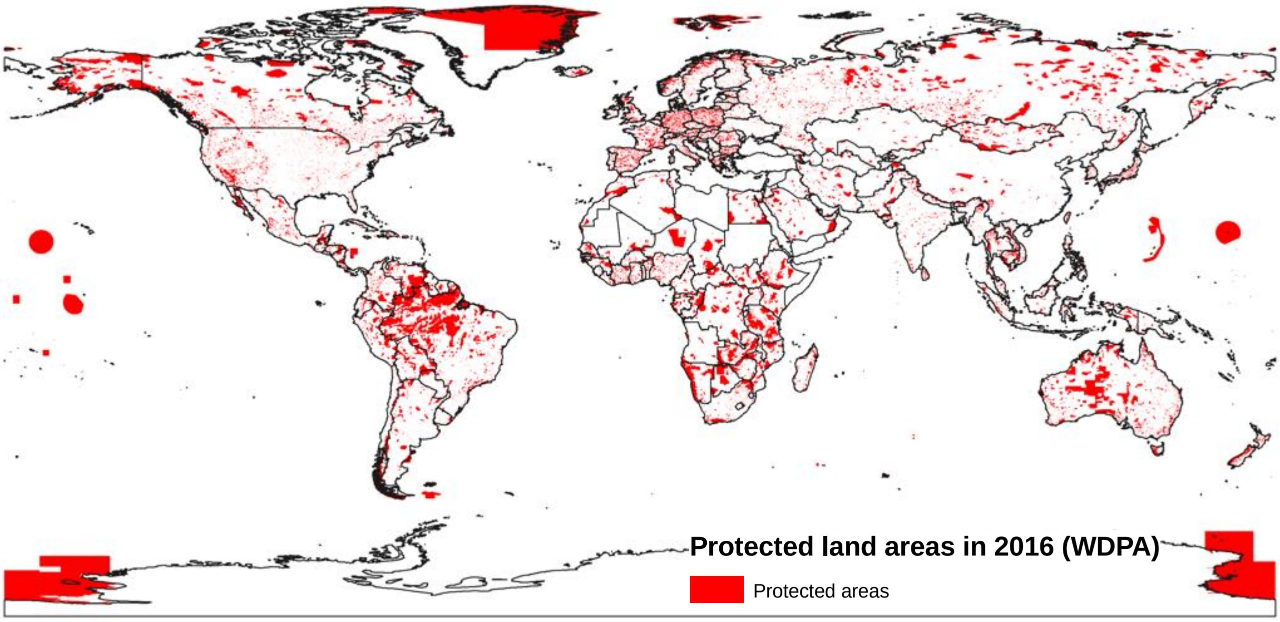

Figure S7. Global map of officially protected land and coastal areas.

Figure S8 presents global suitability of land for plants growth, based on the biophysical constraints listed in Supplementary Methods Table S3, namely soil-related constraints as sourced from the Harmonized World Soil Database [4].

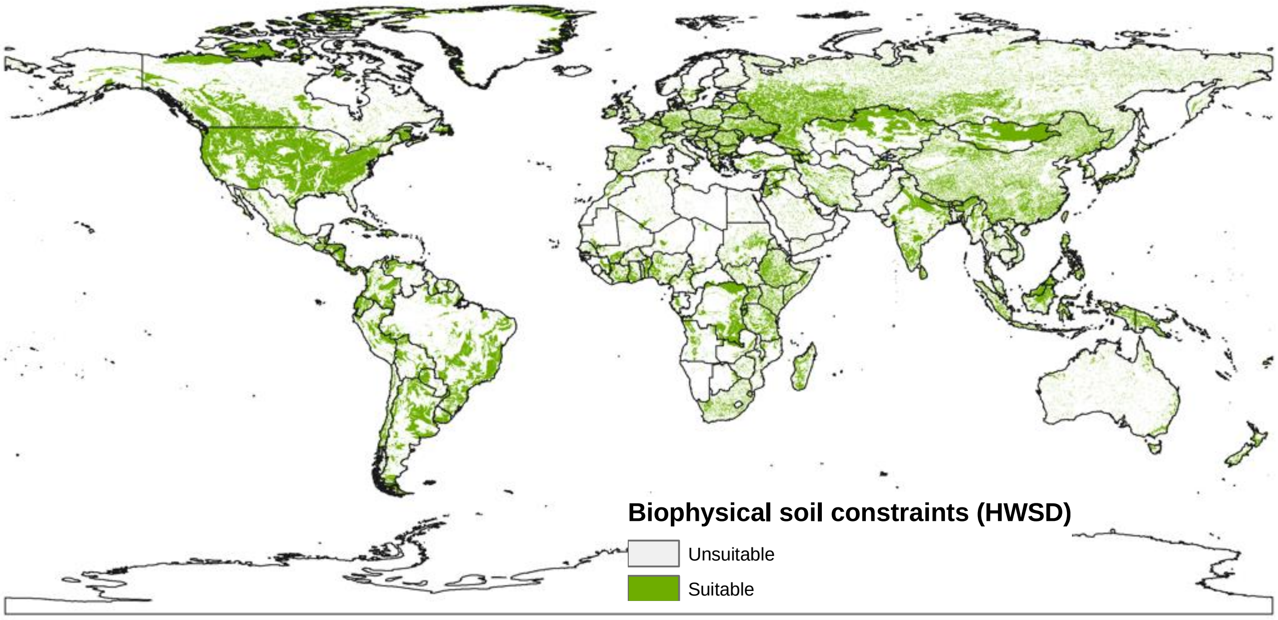

Figure S8. Global map of biophysical soil constraints to plants.

Figure S9 presents the global ecological zones as classified in FAO's Global Ecological Zones (GEZ) [5].

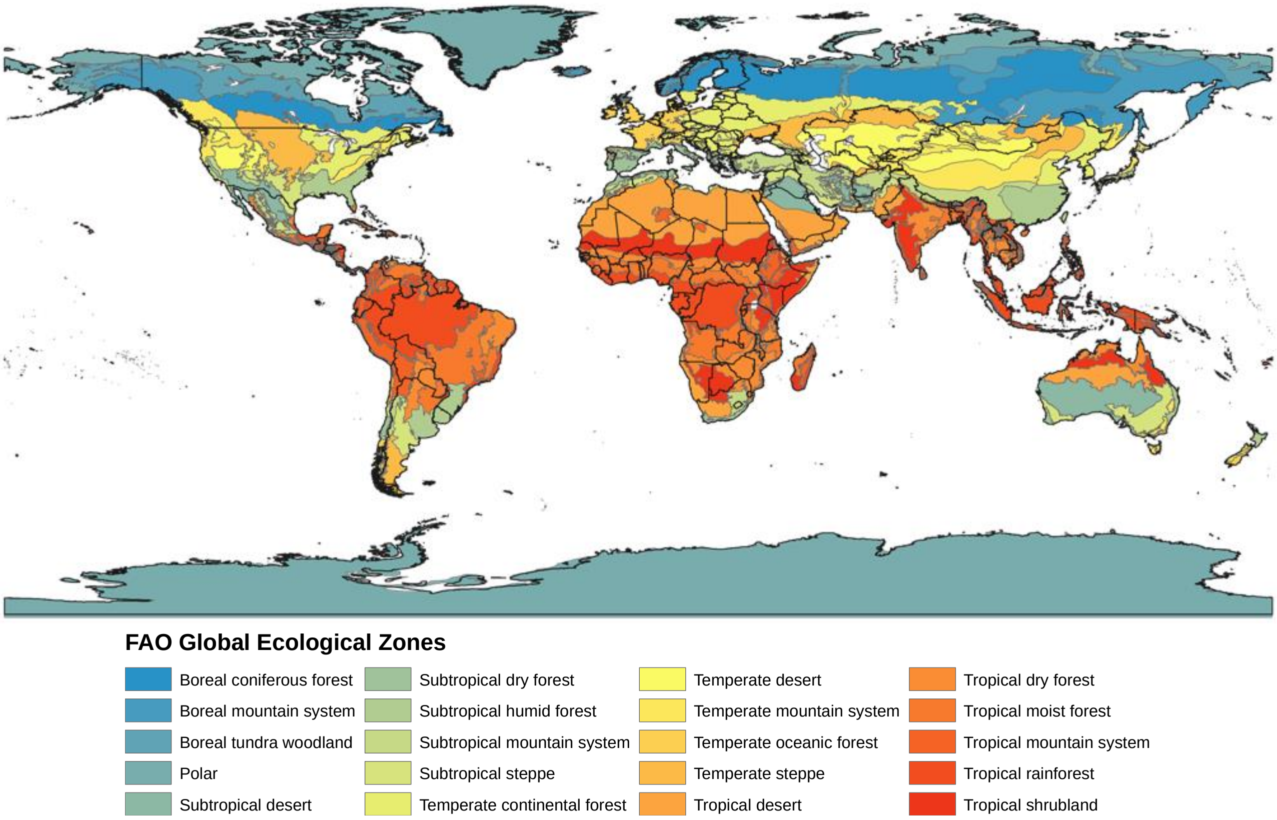

Figure S9. Global map of FAO's Global Ecological Zones.

Figure S10 shows the world regions provided by Esri ArcGIS Data & Maps (2020), ArcGIS (https://www.arcgis.com/home/item.html?id=a79a3e4dc55343b08543b1b6133bfb90)

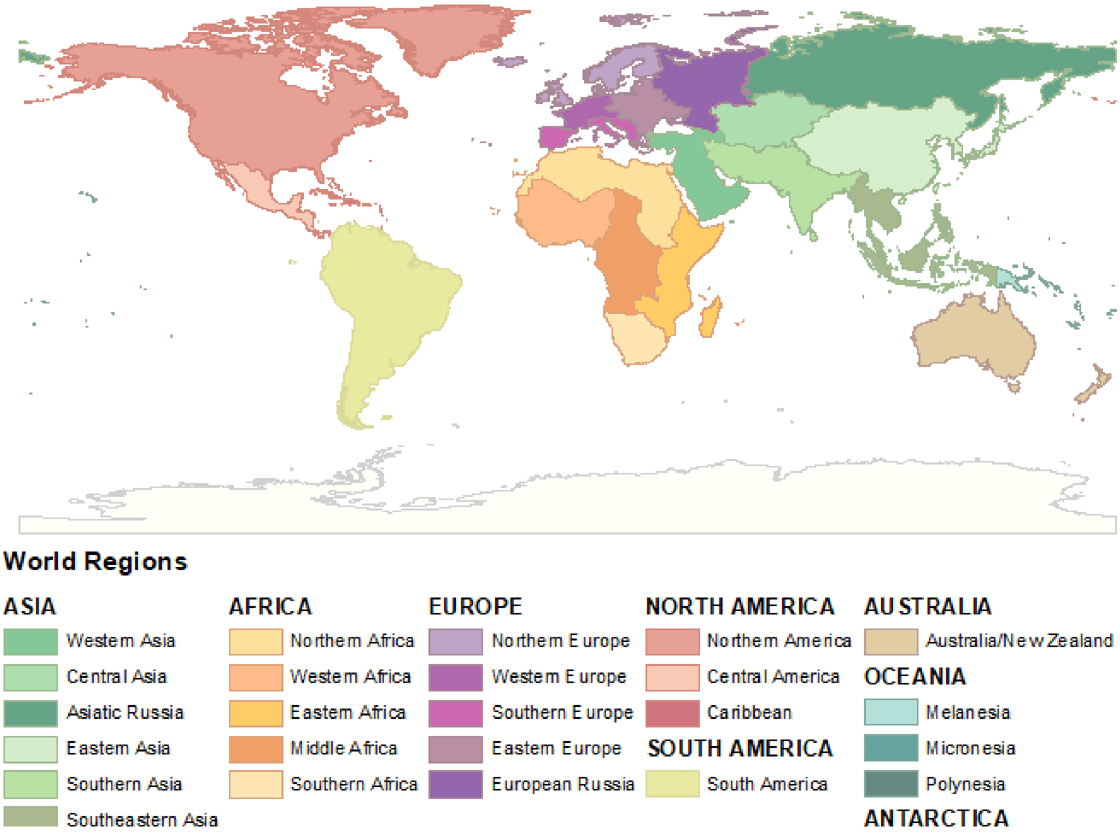

Figure S10. Global map of World Regions

Identified target areas (marginal land)

Table S1 Identified marginal land up to 50 t SOC ha^{-1} (30 cm) per global ecological zone and world regions and corresponding top concentrations

Global ecological zone (GEZ)	Mha per GEZ	% of total area	Top world region per GEZ	n	World region	Mha per world region	% of total area	Top GEZ per region	n
Boreal coniferous forest	6.0E-3	0%	European Russia	2	Asiatic Russia	7.6E-2	0%	Boreal tundra woodland	2
Boreal mountain system	1.6E-3	0%	Eastern Asia	2	Australia/New Zealand	2.0E-1	1%	Tropical desert	7
Boreal tundra woodland	7.0E-2	0%	Asiatic Russia	1	Caribbean	3.6E-4	0%	Tropical moist forest	3
Subtropical desert	2.0E+0	7%	Western Asia	4	Central America	2.4E-3	0%	Subtropical desert	4
Subtropical dry forest	9.2E-2	0%	Northern Africa	10	Central Asia	3.9E+0	14%	Temperate desert	4
Subtropical humid forest	1.3E-2	0%	Northern America	5	Eastern Africa	5.2E-1	2%	Tropical shrubland	4
Subtropical mountain system	1.3E+0	5%	Southern Asia	9	Eastern Asia	1.1E+1	39%	Temperate desert	6
Subtropical steppe	2.6E+0	10%	Southern Asia	6	Eastern Europe	2.4E-3	0%	Temperate continental forest	3
Temperate continental forest	3.0E-2	0%	Western Asia	6	European Russia	2.6E-2	0%	Temperate steppe	4
Temperate desert	1.1E+1	40%	Eastern Asia	7	Middle Africa	2.6E-1	1%	Tropical mountain system	6
Temperate mountain system	2.3E+0	8%	Eastern Asia	8	Northern Africa	2.8E+0	10%	Tropical shrubland	8
Temperate oceanic forest	1.9E-3	0%	Western Europe	4	Northern America	1.9E-1	1%	Temperate desert	8
Temperate steppe	1.7E+0	6%	Eastern Asia	5	Northern Europe	8.5E-4	0%	Temperate oceanic forest	3
Tropical desert	3.4E+0	13%	Northern Africa	9	South America	1.3E+0	5%	Tropical mountain system	8
Tropical dry forest	3.5E-2	0%	South America	10	Southeastern Asia	2.3E-4	0%	Tropical dry forest	1
Tropical moist forest	1.5E-2	0%	South America	7	Southern Africa	3.8E-1	1%	Tropical desert	6
Tropical mountain system	6.1E-1	2%	South America	7	Southern Asia	2.9E+0	11%	Subtropical steppe	13
Tropical rainforest	5.7E-3	0%	Southern Asia	6	Southern Europe	2.5E-2	0%	Subtropical dry forest	5
Tropical shrubland	2.1E+0	8%	Northern Africa	8	Western Africa	9.7E-1	4%	Tropical desert	8
N/A	N/A		N/A		Western Asia	2.9E+0	11%	Subtropical desert	10
N/A	N/A		N/A		Western Europe	1.1E-3	0%	Temperate oceanic forest	3
Grand total	2.7E+1	100%		116		2.7E+1	100%		116

Table S2. **Top target area-biopump matches, area availability, and changes in soil organic carbon (SOC) between the initial (year 2020) and final (year 2100) stock levels, including SOC losses from erosion and net SOC; sorted by net SOC**

	World region	Global Ecological Zones	Plant species	**Area**	Initial SOC	Final SOC	SOC change (final/initial)	Eroded SOC	**Net SOC (final-eroded)**	Net SOC change (net/final)
					2020	2100	2100	2100	**2100**	2100
				**[Σha]**	[t C/ha]	[t C/ha]	[%]	[t C/ha]	**[t C/ha]**	[%]
1	Australia/New Zealand	Tropical dry forest	Melia azedarach var. australasica	3258	25.57	170.48	667%	-12.43	**158.04**	93%
2	Northern Africa	Tropical dry forest	Melia azedarach	1053	28.87	152.13	527%	-7.26	**144.87**	95%
3	Eastern Africa	Tropical dry forest	Cannabis sativa ssp. indica	2097	49.25	170.36	346%	-34.79	**135.57**	80%
4	Central America	Tropical dry forest	Cannabis sativa ssp. indica	468	40.63	135.97	335%	-17.47	**118.50**	87%
5	Eastern Africa	Tropical mountain system	Cannabis sativa ssp. indica	2601	36.23	134.49	371%	-59.93	**74.56**	55%
6	European Russia	Boreal coniferous forest	Silphium perfoliatum L.	5904	48.95	70.49	144%	-0.56	**69.92**	99%
7	European Russia	Boreal mountain system	Silphium perfoliatum L.	234	48.47	69.60	144%	-0.04	**69.56**	100%
8	Northern Europe	Temperate continental forest	Silphium perfoliatum L.	261	47.25	64.99	138%	-1.49	**63.50**	98%
9	Northern Europe	Boreal coniferous forest	Silphium perfoliatum L.	117	42.38	62.33	147%	-0.60	**61.74**	99%
10	Southeastern Asia	Tropical dry forest	Melia azedarach var. australasica	225	37.44	154.26	412%	-108.21	**46.05**	30%
11	Northern Africa	Tropical moist forest	Zea mays ssp. mays	18	28.84	54.70	190%	-9.13	**45.57**	83%
12	Northern Europe	Temperate oceanic forest	Silphium perfoliatum L.	468	31.31	45.35	145%	-2.32	**43.03**	95%
13	Eastern Europe	Temperate mountain system	Silphium perfoliatum L.	207	40.75	45.40	111%	-5.37	**40.03**	88%
14	Western Europe	Temperate mountain system	Silphium perfoliatum L.	9	30.48	34.62	114%	-0.41	**34.21**	99%
15	Central America	Tropical moist forest	Musa acumita	81	46.37	55.68	120%	-24.19	**31.49**	57%
16	Western Europe	Temperate oceanic forest	Silphium perfoliatum L.	828	34.60	38.63	112%	-7.38	**31.25**	81%
17	Australia/New Zealand	Tropical shrubland	Zea mays ssp. mays	44766	31.55	60.18	191%	-30.72	**29.45**	49%
18	Central America	Tropical rainforest	Musa salaccensis	558	42.50	58.98	139%	-33.20	**25.77**	44%
19	Australia/New Zealand	Tropical desert	Acacia erioloba	154422	30.99	25.99	84%	-10.73	**15.26**	59%
20	Australia/New Zealand	Subtropical steppe	Jatropha curcas	504	37.40	17.10	46%	-3.86	**13.25**	77%
21	Western Africa	Tropical moist forest	Crotalaria juncea	2385	29.65	49.97	169%	-38.40	**11.56**	23%
22	Western Africa	Tropical shrubland	Populus euphratica	302508	26.90	29.43	109%	-20.78	**8.65**	29%
23	Middle Africa	Tropical moist forest	Paspalum plicatulum	81	44.61	25.81	58%	-17.68	**8.13**	31%
24	Australia/New Zealand	Temperate oceanic forest	Silphium perfoliatum L.	441	44.26	45.79	103%	-40.47	**5.31**	12%
25	South America	Tropical moist forest	Crotalaria lanceolata	7182	40.03	45.16	113%	-40.97	**4.20**	9%
26	South America	Tropical dry forest	Miscanthus spp.	22644	33.44	18.97	57%	-16.39	**2.57**	14%

Potential SOC changes associated with plant species and land use change

Computed values from datasets provided in [6] are presented in Table S3. These values are used to evaluate the potential biopumps for our case study, among other databases. Current land use is perennial crops and all SOC changes emerged from previous land uses corresponding to annual crops, fallow land, grassland, and forest land. The temporal consideration from a previous land to perennial crop is at least one year.

Table S3. Mean annual soil organic carbon stock changes (ΔSOC) and standard deviation (SD) per perennial crop, climate type (tropical, subtropical, and temperate), land use change (annual crop, fallow, grassland, and forest land to perennial crop), topsoil (0-30 cm), subsoil (30-100).

Common name	Crop type	Climate type	Soil depth limit (cm)		Count	Mean	SD
			Lower	Higher	(n)	Mg C ha^{-1} yr^{-}	Mg C ha^{-1} yr^{-1}
Conversion annual crop to perennial							
Acerola	woody	Tropical	0	20	1	0.21	
Banana	herbaceous	Tropical	0	30	1	0.10	
Brachiaria	herbaceous	Tropical	0	20	6	-0.15	1.36
Cloves	woody	Tropical	0	30	2	0.10	
Cloves	woody	Tropical	30	100	6	-0.13	0.11
Coconut	woody	Tropical	0	20	2	0.07	
Eucalyptus	woody	Tropical	0	30	2	1.01	
Eucalyptus	woody	Tropical	30	100	6	0.12	0.27
Guava	woody	Tropical	0	30	4	0.07	0.22
Guava	woody	Tropical	30	100	6	0.16	0.08
Litchi	woody	Tropical	0	30	2	0.19	
Litchi	woody	Tropical	30	100	6	-0.08	0.12
Mango	woody	Tropical	0	30	2	-0.01	
Mango	woody	Tropical	30	100	6	-0.03	0.05
Prosopis	woody	Tropical	0	30	2	-0.09	
Prosopis	woody	Tropical	30	100	6	-0.07	0.23
Sugarcane	herbaceous	Tropical	0	30	7	0.33	0.53
Sugarcane	herbaceous	Tropical	30	100	2	-0.61	
Tea	woody	Tropical	30	100	3	-0.80	1.07
Black locust	woody	Temperate	0	30	3	0.01	0.01
Black locust	woody	Temperate	30	100	1	-0.01	
Miscanthus	herbaceous	Temperate	0	30	39	0.39	3.13
Miscanthus	herbaceous	Temperate	30	100	48	0.08	1.02
Poplar	woody	Temperate	0	30	34	0.08	0.44
Poplar	woody	Temperate	30	100	19	-0.02	0.26
Poplar-Willow	woody	Temperate	0	30	6	0.13	0.13
Poplar-Willow	woody	Temperate	30	100	1	0.38	
Spruce	woody	Temperate	0	30	1	0.11	
Switchgrass	herbaceous	Temperate	0	30	55	0.31	1.35
Switchgrass	herbaceous	Temperate	30	100	37	0.76	2.65
Willow	woody	Temperate	0	30	36	0.59	4.20
Willow	woody	Temperate	30	100	38	0.72	3.95
Acacia	woody	Subtropical	0	15	1	0.66	
Almond	woody	Subtropical	0	15	2	3.56	
Bahiagrass	herbaceous	Subtropical	0	20	1	0.23	
Black locust	woody	Subtropical	0	30	3	0.39	0.40
Black locust	woody	Subtropical	30	100	2	0.03	
Cotton	woody	Subtropical	0	20	4	-0.22	0.41
Eucalyptus	woody	Subtropical	0	30	8	0.05	0.19
Giant reed	herbaceous	Subtropical	0	30	3	0.24	0.37
Giant reed	herbaceous	Subtropical	30	100	3	0.14	0.14
Miscanthus	herbaceous	Subtropical	0	30	4	0.33	0.36
Miscanthus	herbaceous	Subtropical	30	100	2	0.11	
Peanut	herbaceous	Temperate	0	20	2	0.85	
Poplar	woody	Subtropical	0	30	5	-0.49	1.09

Poplar	woody	Subtropical	30	100	2	0.04	
Ramie	herbaceous	Subtropical	0	30	4	0.45	0.07
Switchgrass	herbaceous	Subtropical	0	30	2	0.03	
Switchgrass	herbaceous	Subtropical	30	100	2	0.11	
Willow	woody	Subtropical	0	30	2	0.29	
Willow	woody	Subtropical	30	100	2	0.00	
Conversion fallow crop to perennial							
Casuarina	woody	Tropical	0	30	1	-4.75	
Eucalyptus	woody	Tropical	0	30	1	-3.67	
Eucalyptus	woody	Tropical	30	100	1	-3.71	
Acacia	woody	Subtropical	0	15	5	0.62	0.67
Almond	woody	Subtropical	0	15	6	-2.04	2.09
Poplar	woody	Subtropical	0	15	1	0.21	
Vineyard	woody	Subtropical	0	10	2	0.49	
Conversion grassland to perennial							
Cocoa	woody	Tropical	0	10	3	0.71	0.78
Coffee	woody	Tropical	0	30	26	-0.05	0.78
Coffee	woody	Tropical	30	100	15	0.40	3.40
Palm.Banana.others	herbaceous	Tropical	0	30	3	-0.54	0.80
Sugarcane	herbaceous	Tropical	0	30	52	-0.67	1.30
Sugarcane	herbaceous	Tropical	30	100	102	-0.59	2.23
Atriplex	woody	Temperate	0	30	3	0.09	0.35
Atriplex	woody	Temperate	30	200	4	0.73	0.53
Miscanthus	herbaceous	Temperate	0	30	20	0.50	3.04
Miscanthus	herbaceous	Temperate	30	100	12	0.06	0.46
Poplar	woody	Temperate	0	30	4	-0.50	0.29
Poplar	woody	Temperate	30	100	3	0.41	0.53
Switchgrass	herbaceous	Temperate	0	30	14	0.07	2.08
Switchgrass	herbaceous	Temperate	30	100	5	-1.61	1.70
Willow	woody	Temperate	0	30	13	-0.82	3.93
Willow	woody	Temperate	30	100	23	0.69	2.26
Cotton	woody	Subtropical	0	20	4	-0.45	0.42
Miscanthus	herbaceous	Subtropical	0	30	2	1.59	
Conversion forest land to perennial							
Annona	woody	Tropical	0	30	1	-0.52	
Annona	woody	Tropical	30	100	1	-0.28	
Araucaria	woody	Tropical	0	30	1	1.62	
Araucaria	woody	Tropical	30	100	1	0.68	
Banana	herbaceous	Tropical	0	30	4	-0.79	1.55
Banana	herbaceous	Tropical	30	100	1	-1.40	
Brachiaria	herbaceous	Tropical	0	30	6	-0.36	1.05
Cashew	woody	Tropical	0	30	1	-1.02	
Cashew	woody	Tropical	30	100	1	-0.57	
Cedar	woody	Tropical	0	30	2	-1.74	
Cedar	woody	Tropical	30	100	2	-0.94	
Cocoa	woody	Tropical	0	30	15	-0.65	2.25
Cocoa	woody	Tropical	30	100	8	0.07	0.25
Coffee	woody	Tropical	0	30	9	-0.73	0.54
Coffee	woody	Tropical	30	100	7	-0.20	0.28
Cotton.corn.groundnut	woody	Tropical	0	30	12	-0.03	0.03
Eucalyptus	woody	Tropical	0	30	3	-0.08	0.05
Eucalyptus	woody	Tropical	30	100	3	0.61	0.14
Fruit	woody	Tropical	30	100	1	-0.35	
Guava	woody	Tropical	0	30	1	-0.88	
Guava	woody	Tropical	30	100	1	-0.20	
Mango	woody	Tropical	0	30	1	-1.05	
Mango	woody	Tropical	30	100	1	-0.58	
Manilkara	woody	Tropical	0	30	1	-0.82	
Manilkara	woody	Tropical	30	100	1	-0.43	
PalmOil	herbaceous	Tropical	0	30	2	5.90	
Pinus	woody	Tropical	0	30	1	2.76	
Pinus	woody	Tropical	30	100	1	0.10	
Rubber	woody	Tropical	0	30	1	-0.29	
Sugarcane	herbaceous	Tropical	0	30	6	-1.98	2.38

Sugarcane	herbaceous	Tropical	30	100	11	-1.97	2.82
Tea	woody	Tropical	0	30	72	-0.11	0.22
Tea	woody	Tropical	30	100	35	-0.23	0.23
Annato	woody	Tropical	0	30	2	-0.36	
Annato	woody	Tropical	30	100	4	0.04	0.07
Banana	herbaceous	Tropical	0	30	1	-0.78	
Brazil_nut	woody	Tropical	0	30	2	0.04	
Brazil_nut	woody	Tropical	30	100	4	0.08	0.05
Cocoa	woody	Tropical	0	30	4	-1.02	1.20
Cocoa	woody	Tropical	30	100	3	-0.01	0.05
Coffee	woody	Tropical	0	30	2	-1.27	
Coffee	woody	Tropical	30	100	3	-0.03	0.37
Cupuacu	woody	Tropical	0	30	2	-0.18	
Cupuacu	woody	Tropical	30	100	4	0.07	0.05
Eucalyptus	woody	Tropical	0	30	1	0.55	
Eucalyptus	woody	Tropical	30	100	1	-0.45	
Jatropha	woody	Tropical	0	30	4	-4.36	5.06
PalmOil	herbaceous	Tropical	0	30	1	4.62	
Peach_palm_fruit	woody	Tropical	0	30	4	-0.32	0.68
Peach_palm_fruit	woody	Tropical	30	100	8	0.03	0.09
Rubber	woody	Tropical	0	30	4	-0.03	0.06
Rubber	woody	Tropical	30	100	4	0.04	0.09
Poplar	woody	Temperate	0	30	2	0.85	
Olive	woody	Subtropical	0	30	2	1.45	
Conversion short rotation coppice to perennial crops							
Maize	herbaceous	Temperate	0	30	9	0.33	2.74
Miscanthus	herbaceous	Temperate	0	30	1	-3.02	
Miscanthus	herbaceous	Temperate	30	100	2	-0.63	
Ryegrass	herbaceous	Temperate	0	30	9	1.22	1.79

Figure S11 summarises the assessed annual SOC changes per climate type (here tropical, subtropical, and temperate) and per woody and herbaceous crop type. Note that the positive values from the conversion from forestry to perennial species corresponds to woody species (more precisely related with olive and poplar) in subtropical and temperate climate types, referring to reported values (only 2 counts) from land use change over 14 to 16 years.

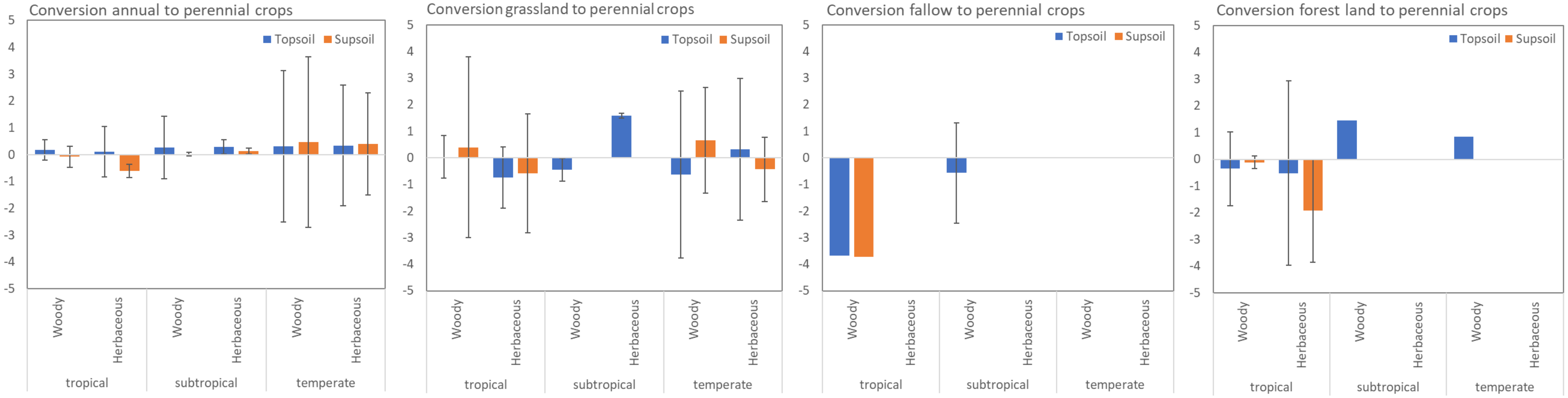

Figure S11. Mean annual soil organic carbon stock changes under perennial woody and herbaceous crops per climate type.

References

[1] FAO and ITPS, Global Soil Organic Map V1.5: Technical Report, Rome: Food and Agriculture Organization of the United Nations, 2020. https://doi.org/https://doi.org/10.4060/ca7597en.

[2] A. Di Gregorio, Land Cover Classification System. Classification concepts. Software version 3, Rome: Food and Agriculture Organization of the United Nations, 2016.

[3] UNEP-WCMC, User Manual for the World Database on Protected Areas and world database on other effective area- based conservation measures : 1.6, UNEP-WCMC: Cambridge, UK., 2019. https://wdpa.s3-eu-west-1.amazonaws.com/WDPA_Manual/English/WDPA_WDOECM_Manual_1_6.pdf.

[4] FAO/IIASA, Harmonized World Soil Database (version 1.2), FAO, Rome, Italy and IIASA, Laxenburg, Austria, 2009.

[5] FAO, Global ecological zones for FAO forest reporting: 2010 Update, Rome: Food and Agriculture Organization of the United Nations, 2012.

[6] A. Ledo, J. Hillier, P. Smith, E. Aguilera, S. Blagodatskiy, F.Q. Brearley, A. Datta, E. Diaz-Pines, A. Don, M. Dondini, J. Dunn, D.M. Feliciano, M.A. Liebig, R. Lang, M. Llorente, Y.L. Zinn, N. McNamara, S. Ogle, Z. Qin, P. Rovira, R. Rowe, J.L. Vicente-Vicente, J. Whitaker, Q. Yue, A. Zerihun, A global, empirical, harmonised dataset of soil organic carbon changes under perennial crops, Sci. Data. 6 (2019) 1–7. https://doi.org/10.1038/s41597-019-0062-1.
